# Human germline and pan-cancer variomes and their distinct functional profiles

**DOI:** 10.1093/nar/gku772

**Published:** 2014-09-17

**Authors:** Yang Pan, Konstantinos Karagiannis, Haichen Zhang, Hayley Dingerdissen, Amirhossein Shamsaddini, Quan Wan, Vahan Simonyan, Raja Mazumder

**Affiliations:** 1 The Department of Biochemistry & Molecular Medicine, George Washington University Medical Center, Washington, DC 20037, USA; 2 Center for Biologics Evaluation and Research, US Food and Drug Administration, 10903 New Hampshire Avenue, Silver Spring, MD 20993, USA; 3 McCormick Genomic and Proteomic Center, George Washington University, Washington, DC 20037, USA

## Abstract

Identification of non-synonymous single nucleotide variations (nsSNVs) has exponentially increased due to advances in Next-Generation Sequencing technologies. The functional impacts of these variations have been difficult to ascertain because the corresponding knowledge about sequence functional sites is quite fragmented. It is clear that mapping of variations to sequence functional features can help us better understand the pathophysiological role of variations. In this study, we investigated the effect of nsSNVs on more than 17 common types of post-translational modification (PTM) sites, active sites and binding sites. Out of 1 705 285 distinct nsSNVs on 259 216 functional sites we identified 38 549 variations that significantly affect 10 major functional sites. Furthermore, we found distinct patterns of site disruptions due to germline and somatic nsSNVs. Pan-cancer analysis across 12 different cancer types led to the identification of 51 genes with 106 nsSNV affected functional sites found in 3 or more cancer types. 13 of the 51 genes overlap with previously identified Significantly Mutated Genes (Nature. 2013 Oct 17;502(7471)). 62 mutations in these 13 genes affecting functional sites such as DNA, ATP binding and various PTM sites occur across several cancers and can be prioritized for additional validation and investigations.

## INTRODUCTION

Non-synonymous single nucleotide variation (nsSNV) can have profound effects on protein function because of resulting changes to the amino acid sequence of the protein (1–4). By knowing the distribution of all nsSNVs on the human proteome, the number of expected variations in a specific protein can be calculated (5). Additionally, mapping of disease-related and functional information onto the proteome can provide a comprehensive view of the impact of nsSNVs (2,6–7). There are several databases that contain SNV data and disease-related annotations (OMIM (8), ClinVar (9), SwissVar (10), LSDB (11), HGMD (6), dbSNP (12)). At the same time, Next-Generation Sequencing (NGS) technology is rapidly becoming a mainstream approach for identifying thousands of novel mutations and polymorphisms through national and international collaborative projects like the 1000 Genome Project (13), TCGA project (http://cancergenome.nih.gov/), NCI-60 panel project (14) and others (15,16). There are not yet standardized methods for NGS analysis, so there is a high level of variability between different analysis pipelines (17). Thus, there is a developing need for the creation of secondary curated databases to provide mechanisms for biocuration and standardization of NGS analysis pipelines and comparisons of their results (3,18–19).

To take advantage of the vast volume of human variation data accumulated over the last few years, the integration and unification of the data is critical, as a comprehensive data set is necessary to better summarize the variation trends. Based on our previous work (2,5,20), we have mapped and analyzed nsSNV impact in terms of N-linked glycosylation and active sites of enzymes for single-nucleotide polymorphisms (SNPs) available in dbSNP (12), somatic mutations from COSMIC (21) and variations reported in UniProtKB (22). To ensure a comprehensive study, we have now further extended our non-redundant nsSNV data set to cover all somatic mutations published by TCGA, ICGC (15) and IntOGen (23) as well as cancer-related mutations from NCI-60 cell lines and ongoing Curated Short Reads (CSR) (3) project. All variations were mapped to functional sites and unified based on the UniProtKB/Swiss-Prot defined complete human proteome (22) while different disease annotations were labeled and unified using Disease Ontology (DO) terms (24) for comparative analysis purposes.

Protein function has been observed to rely on select essential sites instead of requiring all sites to be indispensable. Many of these sites are crucial for normal physiological functions and are categorized according to function as active sites, binding sites, post-translational modification (PTM) sites, etc. An enzyme active site is the region where substrates bind to and are catalyzed by an enzyme, but the exact definition can differ across databases and publications (2,25–27). Binding sites, on the other hand, exist not only in enzymes but in any protein that interacts with other biological components in a cell. Both UniProtKB/Swiss-Prot (22) and NCBI Conserved Domain Database (CDD) (26) provide rich annotation on active sites and binding sites of human proteins. CDD stores a significant amount of binding site annotations about small molecules like ligands and ions as well as macromolecular protein complexes. PTMs have always been considered important for their role in control of protein functionality and activity under different physiological conditions (28) and they have been extensively studied in various species (29). With the advent of new and powerful technologies, hundreds of thousands of diverse PTMs have been identified from organisms ranging from prokaryotes to eukaryotes. Cutting edge PTM research includes understanding the biological functions of PTMs (30), associating PTM to traits and the interplay and roles of different types of modifications (28,31–32). There exist several databases that store PTM-related information. Several efforts have been made to resolve this heterogeneity and to organize the data in a better way. A controlled vocabulary has been developed by UniProKB/Swiss-Prot with more than 100 types of PTMs annotated in the feature (FT) line. Annotations from CDD use a similar but different vocabulary. Besides these two major protein annotation sources, dbPTM 3.0 (33) carries out a comprehensive compilation of publicly available databases and generates a data set containing only experimentally verified entries, which include data from UniProtKB/Swiss-Prot, Phospho.ELM (34), PhosphoSitePlus (35), O-GLYCBASE (36), dbSNO (37), SysPTM (38) and HPRD (39). However, heterogeneity issues still left unresolved include (i) annotations are not mapped to a unified proteome reference and (ii) naming of PTMs is not standardized. In this study, we integrated and curated data from all of the above data sources and mapped the functional sites to the UniProtKB/Swiss-Prot human proteome to allow comprehensive and uniform analysis of the effects of germline and somatic nsSNVs.

In contrast to the extensive research performed on the functional impacts of variation (1,5,30,40) or on interpreting the possible function of the PTM/active/binding sites (2,28,31–32), combined studies, though of great importance, are relatively rare. Recently, however, publications on pan-cancer analysis showcase the importance of these types of studies. Examples include studies by Jia *et al.* which analyzed somatic mutations in nine major cancers and resulted in the proposal of 3–5 predominant mutational processes that likely underlie each cancer genome (41). Analysis performed by the pan-cancer genome program (42) demonstrates how such analyses may aid in identifying new patterns of drivers for cancer (43–46). Although pan-cancer analysis is being heavily pursued because of the availability of cancer genomics data, to the best of our knowledge, there is no study that systematically profiles the interplay between germline and somatic variants mapped to an array of functional sites to answer the critical questions like how many sites are impacted and what is the possible relationship between nsSNVs and various functional sites. Here, we present a study that provides a detailed view of the human and tumor protein coding variome and their functional effects. Furthermore, a phylogenetic analysis of NGS whole exome sequencing samples of 30 breast cancer tumor patients and cell lines is provided as a demonstration of how this type of functional analysis and patient classification can provide a novel direction in personalized diagnostics and therapeutic research.

## MATERIALS AND METHODS

### Integration of nsSNV data

The comprehensive non-redundant data set of nsSNVs was compiled from the following sources. The Cancer Genome Atlas (TCGA) and CGHub data portal (https://cghub.ucsc.edu/) was used to download TCGA variation data sets. The latest release of International Cancer Genome Consortium (ICGC) (15) was downloaded in January of 2014 from its FTP site. IntOGen (23) variation data was downloaded in January 2014 also. CSR (3) data set from 25 TCGA breast cancer patient samples and 5 breast cancer cell lines from NCI-60 panel was downloaded in January 2014. Mutation data from NCI-60 Panel was retrieved from CellMiner verson 1.4 (47). Additional data was obtained from files generated from SNVDis (5) which integrates data from Catalog Somatic Mutations in Cancer (COSMIC), dbSNP, UniProtKB/Swiss-Prot and nextProt (48). It is known the projects mentioned above may collect data from each other. For example, COSMIC is the portal of somatic data mined from literatures and TCGA release; ICGC is built on TCGA and raw data from partner institutes across all over the world; IntOGen collects data mainly from ICGC (15) and TCGA (46). UniProtKB provides curated protein mutations from literature. All somatic nsSNV data was integrated into BioMuta using previously described methodologies (19) for pan-cancer analysis.

### Integration of functional sites

The complete protein functional site data set includes the curated annotations from UniProtKB/Swiss-Prot (22), CDD (26) and dbPTM 3.0 (33). The UniProtKB/Swiss-Prot complete human proteome was downloaded in January 2014. Active and binding sites were retrieved based on the FT line description. Modification data was extracted using PTMlist which is a controlled vocabulary provided by UniProtKB/Swiss-Prot. The NCBI CDD-based annotations of functional sites was retrieved using BATCH CD-Search (49) against CDART database in January 2014. Customized Perl/Python scripts were used to filter out entries such as domains, repeats and motifs with longer than five consecutive amino acids. Filtered sites were categorized manually into various types of PTM sites, active sites and binding sites with original annotations maintained in a separate column. Other PTM records were adopted based on dbPTM 3.0 (33) which collects PTM data from more than 10 different sources.

### Generating unified and non-redundant data sets

Records with genomic positions and variants were translated and annotated through Seattleseq Annotation pipeline (http://snp.gs.washington.edu/SeattleSeqAnnotation138/). Resulting RefSeq accessions and positions were then mapped to UniProtKB/Swiss-Prot complete human proteome using methods we developed in previous works (5,50). Annotations from NCBI/CDD with Refseq protein accessions were directly translated to UniProtKB/Swiss-Prot using methods described earlier (3,5,50). Other annotations with UniProtKB/Swiss-Prot accessions were integrated into the data set following a validation process that confirms the existence of the wild-type amino acid in UniProtKB/Swiss-Prot entry.

### Protein conserved sites and conservation ratio

Degree of conservation for each functional site and nsSNV was measured by using Basic Local Alignment Search Tool (BLAST) (51) against five mammalian proteomes (*Rattus norvegicus*, *Mus musculus*, *Canis familiari*, *Bos Taurus* and *Equus caballus*) downloaded from UniProtKB in January 2014. The five species are selected based on their proteome quality, completeness and evolutionary relatedness to humans based on Representative Proteome Group algorithm developed earlier by us and collaborators (52). Only best hits with e-value lower than 0.00001 for each species were considered homologs. Conservation was calculated as the number of homologs in which the site is conserved. For each site, if the amino acid was found to be conserved across all five selected mammalian species then it was regarded as ‘conserved’. To facilitate the comparative analysis on conservation status of different types of functional sites being affected by nsSNVs, the conservation ratio was calculated. The conservation ratio of a specific amino acid was determined based on the percent of nsSNV impacted amino acids that were found to be conserved. Significance was calculated based on methods described earlier (5,53) where the expected number of nsSNV impacted conserved functional site is = (total number of conserved nsSNV/total number of nsSNV) × total number of the functional site impacted by nsSNV.

### Mapping nsSNV to functional sites/motifs

**Figure 1.**
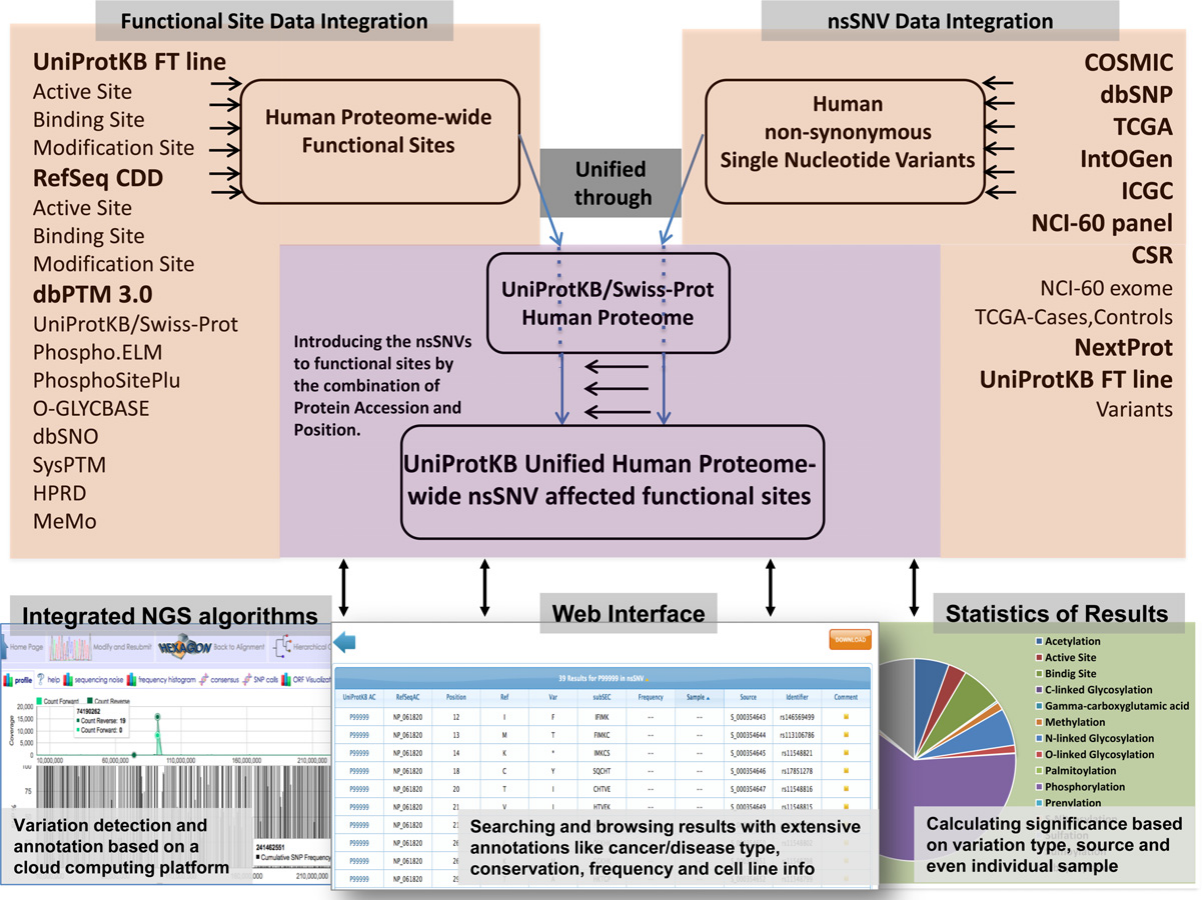
Flowchart of the proteome-wide investigation of the impact of human nsSNV on protein functional sites and the downstream data integration. As shown in the flowchart, once the nsSNV affected functional site is disrupted by alteration, it is then annotated by conservation, NCI-60 cell line mutation and disease annotation.

Customized python scripts were developed to map nsSNVs against functional sites and motifs using specific keys, which is the combination of UniProtKB accession and position and the corresponding variation, as shown in Figure 1. After a variant was successfully mapped to the corresponding functional site, an evaluation process determined whether the variant can affect or cause a loss of function based on the type of functional site (sites/types considered for this study are listed in Table 1). Site information was obtained from the following sources - *Acetylation*: (54–56) *Amidation*: (57) *Biotinylation*: (58) *Crotonylation*: (59) *Gamma-carboxyglutamic acid*: (60,61) *Hydroxylation*: (62) *Methylation*:(63,64) *Myristation*: (65) *N-linked Glycosylation*: (66) O-linked Glycosylation: UniProtKB documents; (66) *C-linked Glycosylation*: UniProtKB documents. *Palmitoylation*: (67,68) *Phosphorylation*: (69–71) *Prenylation*: (72) *S-nitrosylation*: (73,74) *Sulfation*: (75) *Sumoylation*: (76) *Ubiquitylation*: (77). Variants that can be tolerated without potential loss of function, like T to S variation for the third position in the N-linked glycosylation motif NXS/T (where N is an asparagine, X is any amino acid except proline and serine or threonine is the third amino acid), were not regarded as functional site affecting nsSNVs. The final results of this mapping were organized into two files: a file with a list of impacted functional sites and variants with additional annotations such as conservation, frequency, etc.

**Table 1.**
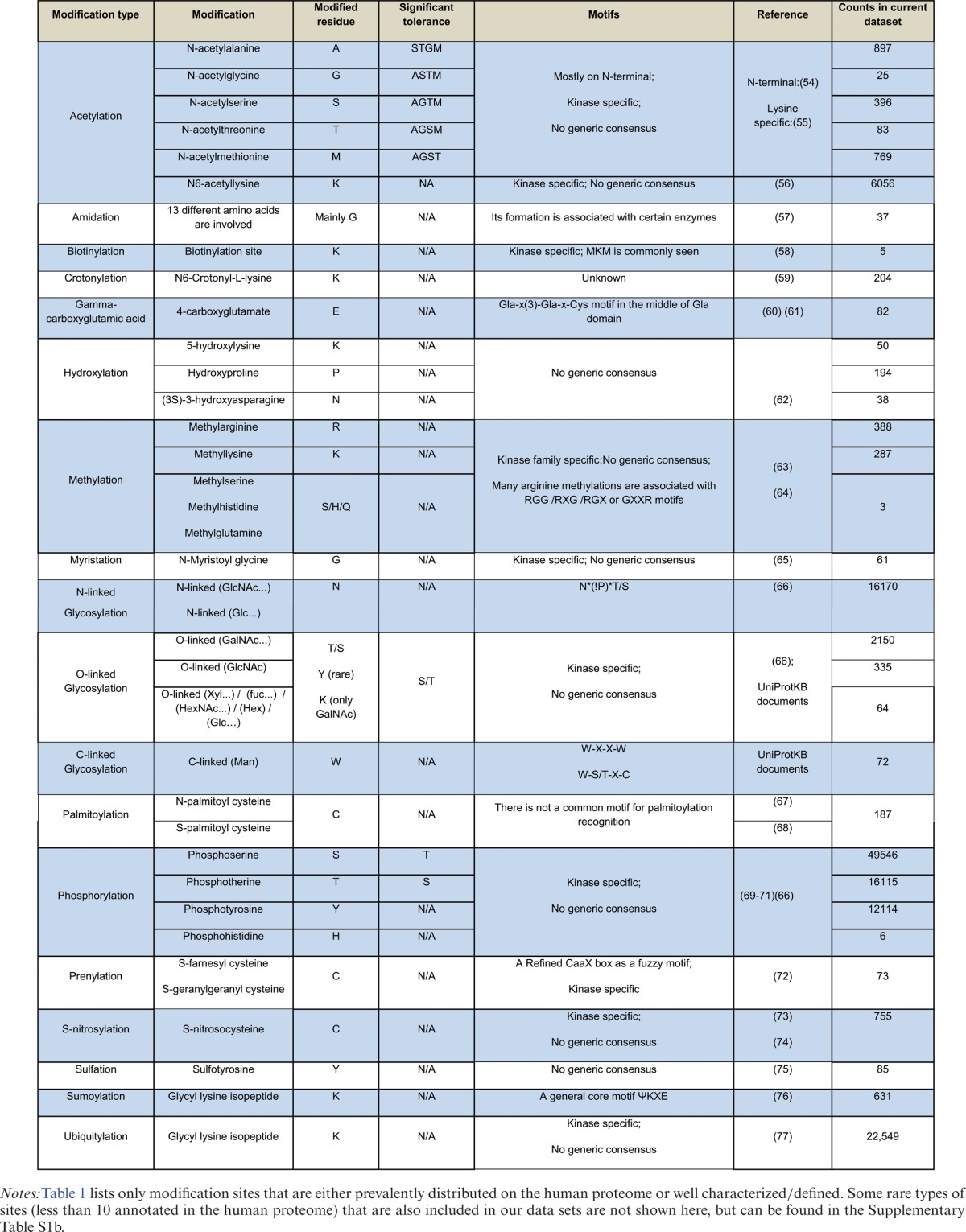
The knowledge summary of 18 common PTM types from the human proteome and their counts in our integrated data set

Significance was calculated based on methods described earlier (5) and represented as the ratio of expected minus observed impact of nsSNVs (53). Therefore, the resultant *P*-value describes the deviance degree between a global ratio and an observed ratio. The calculation of the expected number *n*(*E*) of nsSNVs affecting a certain type of functional site is shown below:

\begin{equation*} \begin{array}{*{20}c} {p(F) = n(F)/L} \\ {n(E) = N*p(F) = N*n(F)/L} \\ \end{array} \end{equation*}

where *N*, *n*(*F*) are total number of variations and the total number of positions for a specific functional site, respectively. The probability *p*(*F*) of observing an amino acid from human proteome as a functional site equals to the value of *n*(*F*) divided by total length of human proteome *L*. Based on the expected number of functional site being affected by nsSNV *n*(*E*), and the actual observed number *n*(*O*), *P*-value is calculated through adopting the Binomial statistic as applied by Mi *et al.* (53)

\begin{equation*} P{\rm -value} = \sum {\left( {\begin{array}{*{20}c} N \\ {n(O)} \\ \end{array}} \right)p(F)^{n(O)} *(1 - p(F))^{(N - n(O))} } \end{equation*}

### Gene Ontology (GO) and pathway analysis

Proteins affected by nsSNVs were analyzed for GO term and pathway enrichment using PANTHER Classification System 9.0 (78,79). In order to identify GO terms and pathways significantly affected, we calculated the *P*-value based on the ratio between nsSNV affected genes and genes that harbor the specific functional site according to UniProtKB/Swiss-Prot annotation of GO terms (5,80).

### Pan-cancer functional site analysis

BioMuta (19) is a curated cancer-centric variation and disease association database in which variations are mapped to the genome/protein/gene. Cancer and somatic mutation data is automatically collected from a variety of data sources including TCGA, ICGC, COSMIC, ClinVar, IntOGen, CSR, UniProt in addition to cancer-associated mutation sites manually extracted from literature. All nsSNVs are mapped to the UniProtKB/Swiss-Prot defined human proteome and associated with UniProtKB accession, position, actual and altered amino acids. A total of 645 706 somatic nsSNVs in BioMuta 2.0 version are associated with at least one cancer type (includes both large-scale studies and small-scale studies). All cancer terms in BioMuta are assigned a DO (24) term to facilitate pan-cancer analysis. All variations were scanned against BioMuta to identify how mutations in different cancer types affect various functional sites. The calculation of significance of observed and expected functional site disruption under each DO term was based on methods described in earlier sections. Heat map and clustering analysis were performed using *heatmap2* function from R package (http://www.R-project.org) and pan-cancer visualization was performed using Circos plots (81).

### SNV-based phylogenetic analysis and group analysis of nsSNV-affected functional sites

The SNV-based phylogenetic analysis using variants called from Whole Exome Sequencing (WXS) data from 25 TCGA breast cancer tumor patients (30 samples) and 5 NCI-60 breast cancer cell lines is based on our previously developed methods (3). SNV-based phylogenetic tree requires two steps, SNV-based genome condensation and alignment followed by phylogenetic tree generation. Alignment of sequences from tumor samples from 25 patients and the NCI-60 cell lines containing a range of genomic sequence around SNVs (0–3) was created using PhyloSNP (3,82). FastTree (83) was used to generate phylogenetic trees with 1000 bootstrap values and a matrix of different sample IDs versus distinct nsSNV-affected functional sites was generated. The Newick format phylogenetic tree was overlaid with functional site information and visualized using Interactive Tree Of Life (ITOL) (84). A clustering of the same group of samples was performed based on their mutational functional site profiles. This profile was built based on the statistical significance of various types of mutation-affected functional site from each sample using the same binomial statistical method mentioned in the early section. For clustering, we only kept those functional site types being impacted by at least one nsSNV across 35 samples. The unsupervised hierarchal clustering is conducted in R package, *heatmap2* function with enabled *hclust* function.

### Disease association data

SwissVar (10) was downloaded in January 2014. Disease association data available through dbSNP including clinical/LSDB variations was downloaded from dbSNP March 2014. The NHGRI GWAS Catalog (85) was integrated based on information obtained in January 2014.

## RESULTS AND DISCUSSION

### Integration and unification of functional sites and nsSNVs

As shown in the flowchart (Figure 1), the very first step includes integration of data from various sources.

The integration process is described in Materials and Methods and the integrated data set is available on the Web (http://hive.biochemistry.gwu.edu/tools/var2function/) for users to browse, search/retrieve and download.

For the nsSNV data set (Supplementary Table S1a), we integrated various germline and somatic variation data sources (COSMIC, ICGC, TCGA, IntOGen, dbSNP, CSR, NCI-60, UniProtKB/Swiss-Prot). For cancer-centric somatic variation databases, the percentage of entries that overlap with dbSNP is less than 11% (COSMIC: 7.2%, ICGC: 10.9%, TCGA: 7.6%, IntOGen: 6.6%), which implies that the majority of variations are most likely not germline polymorphisms. For the functional sites data set, we integrated 17 major types of PTM covering 125 subtypes of non-redundant sites from UniProtKB/Swiss-Prot, CDD and dbPTM 3.0. Additionally, we integrated enzyme active sites and binding sites which gave rise to 1125 different sub-terms. Table 1 and Supplementary Table S1b provide details of the sites analyzed in this study.

### nsSNV impact on functional sites

The integration described above resulted in two comprehensive non-redundant UniProtKB/Swiss-Prot human proteome-centric data sets with 1 705 285 and 259 216 nsSNVs and functional sites, respectively. Mapping of protein variations to functional sites generated a list of variants that cause functional site disruption. A total of 38 549 nsSNVs that potentially affect protein functionality through replacing original residues at active sites, binding sites or PTM sites were identified (Table 2a). Particular variations for certain types of PTMs are tolerated without loss of function. The list of tolerated residues can be found in Table 1. Table 2a provides an overview of how nsSNVs obtained from nine different sources impact the various functional sites in the human proteome. Note that 14 of 19 common functional sites have significantly lower (eight) or higher (six) numbers of affected sites than expected by comparison to the overall distribution of nsSNVs along the human proteome. Acetylation, O-linked glycosylation, phosphorylation and ubiquitylation are the top underrepresented PTMs, methylation and N-linked glycosylation are the top overrepresented PTMs.

**Table 2a.** Summary of nsSNV affected functional sites obtained through proteome-wide survey

	COSMIC	TCGA	ICGC	IntOGen	CSR	NCI60	dbSNP	UniProt	nextProt	Total	Expected	Difference	*P*-value
Acetylation	185	322	198	134	15	15	351	65	190	863	1242.877056	-379.8770564	2.30E-30
Active site	487	1051	506	379	27	40	811	247	541	2385	2214.980703	170.0192966	1.83E-04
Amidation	4	1	0	1	1	1	4	1	2	9	5.572089069	3.427910931	1.12E-01
Binding site	3877	8380	4010	3054	253	451	6395	1549	4005	18 681	17131.61374	1549.386262	4.81E-32
C-linked glycosylation	1	1	2	1	0	0	0	0	0	4	10.84298413	-6.842984134	1.68E-02
Crotonylation	26	24	15	16	0	0	10	0	7	52	30.72178838	21.27821162	2.89E-04
Gamma-carboxyglutamic acid	5	13	6	7	0	0	11	20	22	36	12.34895415	23.65104585	3.46E-08
Hydroxylation	13	13	6	7	0	1	17	2	9	41	42.61895153	-1.618951527	4.42E-01
Methylation	78	109	63	71	8	3	61	32	59	224	102.4059613	121.5940387	2.24E-25
Myristation	4	4	1	1	1	0	2	0	4	9	9.788805121	-0.788805121	4.85E-01
N-linked glycosylation	732	1705	843	656	84	125	1997	207	859	4372	2435.15352	1936.84648	4.53E-273
O-linked glycosylation	28	58	35	19	4	8	108	22	52	205	383.8717577	-178.8717577	8.62E-24
Other lipid modification	0	1	0	0	0	0	0	0	0	1	2.861343035	-1.861343035	2.21E-01
Other modified residue	14	32	16	10	2	1	24	7	16	70	46.23327957	23.76672043	6.75E-04
Palmitoylation	3	10	5	3	0	0	8	3	3	20	28.16163935	-8.161639348	6.88E-02
Phosphorylation	1630	3764	1871	1272	180	257	3917	436	1835	9383	11706.50734	-2323.507343	1.28E-110
Prenylation	0	1	0	0	0	0	2	0	0	3	10.99358114	-7.993581136	4.94E-03
S-nitrosylation	15	19	13	11	0	0	43	7	20	75	113.7007364	-38.70073641	7.20E-05
Sulfation	1	4	5	1	0	0	4	5	5	13	12.80074516	0.199254842	5.15E-01
Sumoylation	10	21	7	8	0	2	13	0	8	48	95.02670818	-47.02670818	7.41E-08
Ubiquitylation	449	798	502	302	29	52	841	104	395	2055	3395.811795	-1340.811795	1.18E-136

nsSNVs can be divided into two general categories: germline and somatic. Because germline variations are heritable and thus under relatively higher selection pressure than somatic variations, somatic nsSNVs can be more randomly distributed on an individual genome. Due to their different properties genetic diseases can be commonly categorized as a simple genetic disorder, such as Mendelian diseases which are caused by germline mutations, or a complex genetic disorder, like cancer which is associated with a spectrum of somatic and germline variants. Although there is no perfect method to distinguish between somatic and germline variation, in this study we differentiate variant type based on dbSNP categorization. Effects of germline and somatic variations in functional sites are shown in Table 2b. Active sites and binding sites show the sharpest difference between the two groups of nsSNVs where somatic nsSNVs have a significantly higher frequency of occurrence than expected compared to germline variations. Although several PTMs have higher rates of potential loss of function due to variation, methylation and O-linked glycosylation stand out because of the distinct level of significance in terms of overrepresentation observed for germline versus somatic types of nsSNVs.

**Table 2b.** Variant type-based comparison of nsSNV's impact on different types of functional sites

	Total	+/- (Somatic)^1^	Not in dbSNP (Somatic)	+/- (Germline)^2^	In dbSNP (Germline)
Acetylation	2.30298E-30	-	4.39E-17	-	3.82E-15
Active site	0.00018315	+	1.56E-14	-	8.53E-05
Amidation	0.111861193	+	2.28E-01	+	2.05E-01
Binding site	4.81249E-32	+	2.26E-110	-	7.86E-20
C-linked glycosylation	0.016786568	-	2.44E-01	-	1.09E-02
Crotonylation	0.000289402	+	8.62E-07	-	2.69E-01
Gamma-carboxyglutamic acid	3.45678E-08	+	1.80E-07	+	1.66E-02
Hydroxylation	0.441785011	-	4.85E-01	-	4.90E-01
Methylation	2.24421E-25	+	2.86E-28	+	4.84E-03
Myristation	0.484614874	+	3.47E-01	-	2.26E-01
N-linked glycosylation	4.5346E-273	+	2.09E-118	+	3.95E-163
O-linked glycosylation	8.61577E-24	-	9.79E-22	-	7.98E-06
Other lipid modification	0.220837281	-	5.03E-01	-	3.03E-01
Other modified residue	0.000674978	+	5.24E-04	+	1.67E-01
Palmitoylation	0.0688012	-	1.67E-01	-	1.73E-01
Phosphorylation	1.2829E-110	-	6.85E-66	-	7.78E-47
Prenylation	0.004939609	-	1.22E-02	-	1.64E-01
S-nitrosylation	7.19603E-05	-	2.24E-06	-	2.91E-01
Sulfation	0.514831294	+	3.33E-01	-	3.84E-01
Sumoylation	7.40765E-08	-	2.25E-03	-	8.66914E-07
Ubiquitylation	1.1793E-136	-	1.57E-71	-	1.10915E-61

^1,2^Over- or underrepresentation.

**Figure 2.**
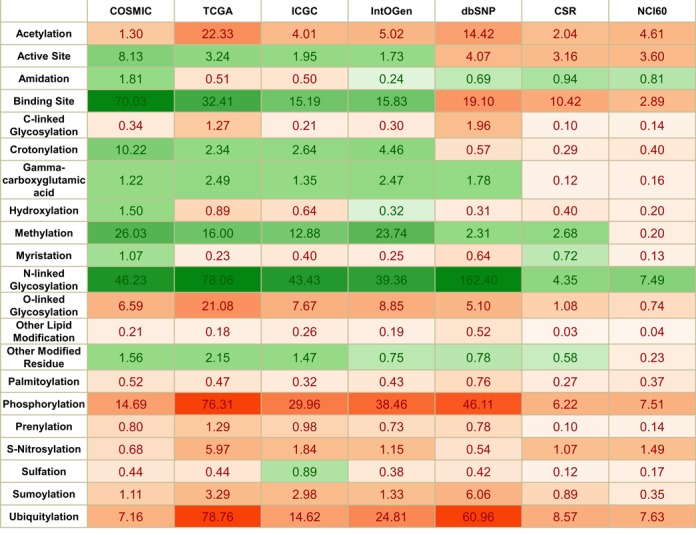
Data source-based analysis of statistical significance of nsSNV affected functional sites. The *P*-value represents the measure of significance of the functional site being affected by nsSNV. For visualization convenience, the calculated *P*-values are then transformed by –log (*P*-value). Overrepresented values are colored as green, and underrepresented values are orange. Darker colors represent a stronger significance.

Based on our findings that the distribution of nsSNVs is not random (2–3,5) we analyzed different data sources to better understand the germline and somatic variome. Table 2b and Figure 2 provides an overview of the analysis results. For Figure 2, green cells represent overrepresentation while red cells show underrepresentation. The intensity of the color is based on *P*-value significance. COSMIC, TCGA, ICGC and IntOGen maintain thousands of cancer-associated variations, most of which are not in dbSNP (more than 92% of COSMIC, 89% of ICGC, 93% of IntOGen, 92% of TCGA) and can be considered somatic mutations. The distribution of nsSNVs from these data sets is similar to each other in terms of their distribution on functional sites. The minor differences observed in Figure 2 among different cancer genomics data portals may be due to the different data sources, collection and analysis methods, and the number of cancer study data sets in these resources. NCI-60 and CSR variants are called from the CSR pipeline, which results in between 80% and 98% of variants currently available in dbSNP for both human tumor samples and cell line samples, explaining the similar pattern they share with dbSNP. Below we provide additional details in terms of GO and pathways that are enriched with genes that have variations mapped to key functional sites (complete list is available in Supplementary Tables S3a and S3b).

**Figure 3.**
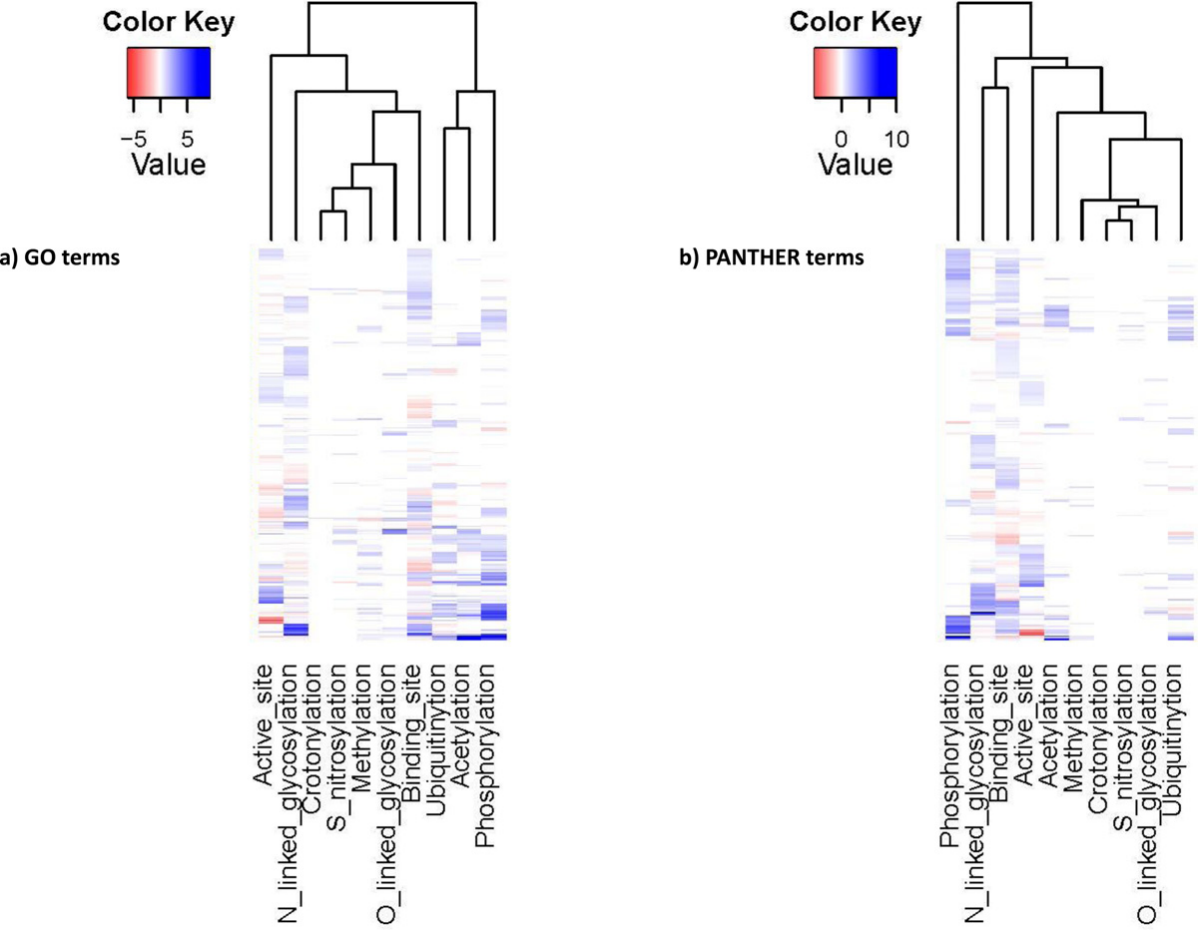
Heatmap and clustering of pathway and GO overrepresentation test. Unique terms are listed in rows and each column represents the degree of impact that a specific type of functional site has on that term. For visualization convenience, the calculated *P*-values are then transformed to –log(*P*-value). Underrepresentation is colored in red and overrepresentation is in blue. The darkness of the color in each box reflects the absolute value of –log(*P*-value). According to the patterns of –log(*P*-value) among various GO terms and PANTHER pathways, functional sites are clustered into different groups.

Phosphorylation sites: Out of 77 734 experimentally verified phosphorylation sites reported on the human proteome, 9383 were replaced by an amino acid residue that cannot be phosphorylated. A total of 5466 of those sites are replaced by nsSNVs not in dbSNP (*P*-values shown in Table 2b and Figure 2). Most of the variants impacted serine (S), which causes the loss of 6063 phosphoserine sites (pS). There were 2033 phosphothreonine (pT) and 1283 phosphotyrosine (pY) sites lost, respectively. Phosphorylation sites have significantly fewer than expected variations (Table 2a, *P*-value: 1.28E-110) for both somatic and germline variations (Table 2b). This is not surprising as phosphorylation is involved in protein activation/deactivation in various pathways including transcription (86), translation (87), cell cycle (88) and signal transduction (89), and is necessary for normal cellular condition such that loss of phosphorylation has been implicated in many diseases (90,91). It is possible that variations that affect phosphorylation sites have a higher chance of being functionally relevant and hence disease causing. Pathway analysis of genes with loss of phosphorylation sites (Figure 3a and b, Supplementary Table S3b) highlights 21 pathways with *P*-value lower than 0.05. The top two overrepresented pathways are angiogenesis (*P*-value 9.12E-06), VEGF signaling pathway (*P*-value 1.90E-05). GO enrichment analysis of genes affected by loss of phosphorylation sites (Figure 3a, Supplementary Table S3a) identified 32 biological processes, 23 molecular function terms having over-/underrepresentation with respect to frequency of nsSNV occurrence, and 9 cellular components terms. A wide variety of biological processes including proteins in metabolic process, cellular process, cell cycle and nucleobase-containing metabolic process are overrepresented, while for molecular functions terms overrepresented terms include kinase activity and nucleic acid binding.

N-linked glycosylation sites: Among 16 170 experimentally verified N-linked glycosylation sites, 4372 nsSNVs alter the NXS/T motif (80), where N is an asparagine, X can be any amino acid except proline and S/T is serine or threonine. Note that 2375 of those impacted N-glycosylation motifs are disrupted by variations not in dbSNP (somatic), the remaining 1997 are germline variations (found in dbSNP) with *P*-values 2.09E-118 and 3.95E-163, respectively (Table 2b and Figure 2). Note that 2387 of the variants disrupt the motif by altering the Ser/Thr residue, while 1938 nsSNVs directly change the asparagine and 70 loss of N-glycosylation motif is caused by a change to proline (P). We observed higher numbers of loss of N-linked glycosylation motifs than expected (*P*-value 4.53E-273, shown in Table 2a) for both somatic and germline nsSNVs (Table 2b), and also across different data sources (Figure 2). This phenomenon suggests that unlike phosphorylation, the N-linked glycosylation landscape is more likely to be altered. These results are counterintuitive to published results by Park *et al.* where they show that N-linked glycosylation motifs are more conserved, unlike what they observed for phosphorylation sites (92). One explanation could be that the two types of PTM sites might be involved in additional yet unknown functions and the two processes deal with loss of functional sites differently. Pathway analysis (Figure 3b, Supplementary Table S3b) highlighted two distinct pathways, which are cadherin signaling pathway (*P*-value 1.55E-03) and Wnt signaling pathway (*P*-value 4.34E-03). GO enrichment analysis (Figure 3a, Supplementary Table S3a) identified 46 terms such as cell adhesion (*P*-value 1.24E-06), nervous system development (*P*-value 7.62E-06) and ectoderm development (*P*-value 6.73E-04).

Ubiquitylation sites: Out of 22 549 experimental verified ubiquitylation sites, 2055 were found to be impacted due to nsSNVs. More than half of those sites (1214) are not in dbSNP (underrepresented, *P*-value 1.57E-71), the rest (841) are affected by nsSNVs found in dbSNP (underrepresented, *P*-value 1.10915E-61) (Tables 2a and 2b and Figure 2). Ubiquitylation sites, like phosphorylation sites, are significantly less altered than what is expected by both germline variation (based on dbSNP records) (Table 2b) and somatic variation (Figure 2). Key ubiquitylation related tasks such as protein degradation, transcriptional regulation and genomic maintenance (93–95) are therefore less prone to variation-related changes.

O-linked glycosylation sites: O-linked glycosylation is important for proprotein processing, biosynthesis of mucins, formation of proteoglycan core proteins and blood group proteins (96,97). Out of 2549 O-linked glycosylation sites in the human proteome, 205 are found to be disrupted. Note that 97 of those impacted O-linked glycosylation site are replaced by nsSNVs not in dbSNP, the remaining 108 are mutated by nsSNVs found in dbSNP (Table 2b and Figure 2). As shown in Table 1, the main type of O-linked glycosylation studied is 2150 O-N-acetylgalactosamine (O-GalNAc) site, out of which 138 are affected by nsSNV. Note that 51 sites out of 335 O-N-acetylglucosamine (O-GlcNAc) sites are changed by nsSNV, some of which can be potentially linked to cross-talk with other PTMs to cause cancer (98). O-linked glycosylation sites unlike N-linked glycosylation sites are significantly underrepresented in terms of the number of proteins that are observed to contain variation at the site versus the number of proteins expected to be impacted (*P*-value 8.62E-24, Table 2a) which underscores the need for studying these two types of glycosylations independently. This underrepresentation trend is true for variations in both dbSNP (Table 2b) and somatic variation data sources (Figure 2), which implies that both cancer-related variations and germline SNPs have a resistance to disruption at the sites to maintain capability of O-linked glycosylation, which also plays role in protein secretion (O-GalNAc) (96) and pathway regulation with phosphorylation (O-GlcNAc) (99,100).

Acetylation sites: Out of 8253 acetylation sites in the human proteome, 863 lose acetylation due to nsSNV. 512 of those are replaced by nsSNVs not in dbSNP, the remaining 351 are mutated by nsSNVs found in dbSNP. Acetylation occurs by two distinct biological mechanisms: enzyme systems, substrate and position preference, which are a co-translational modification termed N-terminal acetylation (101,102); and PTM via Lysine acetylation (103). N-acetylation contributes 2194 sites, 201 of which are affected by nsSNVs. N6-acetyllysine, on the other hand, has 673 sites altered among a total of 6056 sites. According to Tables 2a and 2b, nsSNVs from both germline and somatic variations that cause the loss of acetylation sites are significantly less abundant than expected (*P*-value 4.39E-17 and 3.82E-15, respectively). If one considers the two different types of acetylation, total nsSNV affecting N-acetylation and N6-acetyllysine has similar constraints for variation (*P*-value 1.08226E-14 and 6.03084E-17, respectively). Further investigations into how nsSNVs in dbSNP and those not in dbSNP affect these two types of modification revealed a different type of behavior for the two subtypes of nsSNVs. For nsSNVs in dbSNP, N6-acetyllysine sites are less affected than N-acetylation sites with *P*-value 3.54E-12 to 2.18E-05, while for somatic variations the behavior is opposite (N6-acetyllysine with *P*-value of 2.19E-07 compared to N-acetylation *P*-value 3.68E-11). N6-acetylation is needed for transcriptional regulation and N-acetylation is involved in protein's synthesis, stability and localization (104–106). This indicates that transcriptional regulation is targeted more by somatic mutations.

Methylation sites: Histone methylation as a PTM has been extensively studied (107). Non-histone methylation is prevalent yet relatively poorly understood (108). Methylation can be subtyped according to the modified residue, either lysine (Lys) or arginine (Arg). Subtypes are studied separately as they involve different enzyme families (108–110). Out of 680 experimentally verified methylation sites reported on the human proteome, 224 sites were affected by nsSNV. Note that 163 of those sites were replaced by nsSNVs not in dbSNP, the remaining 61 were found to be mutated by nsSNVs found in dbSNP. There are 388 unique methylarginine sites and 287 unique methyllysine sites in our data set. nsSNV changed 172 arginine sites and 52 lysine sites. From Table 2a, methylation, like N-linked glycosylation, is significantly affected (overrepresentation, *P*-value 2.24E-25) by nsSNV. After subgrouping nsSNVs by somatic and germline origins, a clearer view of how methylation sites are affected at the proteome level was revealed. nsSNVs not in dbSNP show a significant overrepresentation (*P*-value 2.86E-28) compared to germline variations (4.84E-03). Figure 2 shows that loss of methylation site is more prevalent in cancer/somatic mutation centric databases than in dbSNP. The above statistical significance indicates that the PTM methylation is greatly affected in tumor cells which corroborate previous studies linking loss of methylation site to cancer where the authors propose such loss of methylation site with global effects which may alter epigenetic states in a variety of pathologies (111).

Crotonylation sites: Out of 204 experimentally verified crotonylation sites reported in the human proteome, 52 sites are modified by nsSNVs. Note that 42 of these sites are impacted by variations not in dbSNP, the remaining 10 are impacted by polymorphisms found in dbSNP. Loss of crotonylation due to somatic mutations is significantly high (*P*-value 8.62E-07) unlike germline variations (*P*-value 2.69E-01). It was recently shown that lysine crotonylation is a conserved histone PTM found in somatic cells (59). Frequent disruption by somatic mutations of crotonylation sites corroborates previous knowledge that the histone code is more likely to be affected in cancer cells (112).

*S*-nitrosylation sites: Out of 755 experimentally verified S-nitrosylation sites reported in the human proteome, 75 sites are impacted by variation. Note that 32 of those are changed by nsSNVs not in dbSNP, the remaining 43 are affected by nsSNVs found in dbSNP. Somatic variations are underrepresented (*P*-value 2.24E-06). Certain exceptions can be seen from the Figure 2, like COSMIC and IntOGen which provide cancer-centric somatic variation sets that are not significantly underrepresented. As a PTM related to various major diseases (113) including cancer (114), heart diseases (115,116) and Alzheimer's disease (117), it is not clear why S-nitrosylation sites are under functional pressure in cancer cells. Availability of additional experimentally verified S-nitrosylation sites and functional analysis of the loss of nitrosylation sites will provide a better understanding of this PTM.

Enzyme active sites: Out of 14 708 experimental verified active sites reported on the human proteome, 2385 are detected to be substituted by an amino acid residue that can potentially interrupt original activity (2). Note that 1574 of the impacted active sites are affected by somatic nsSNVs, the remaining 811 are from dbSNP. Overall, there appears to be more variation on these sites than expected (Table 2a; *P*-value 1.83E-04). Further comparison between somatic and germline nsSNVs gives a clear indication that active sites are under opposite pressures in terms of significance between the two variation types (Table 1b). Somatic variations have a significantly higher chance of loss of active sites due to variation (*P*-value 1.56E-14) while germline variations usually do not occur at such sites (8.53E-05), a phenomenon we have reported previously (2). As discussed in the previous study (2), nsSNV is assumed to cause a loss or change of function at active or catalytic sites by replacing the essential site with a residue not optimized to the specific interaction required by the enzyme for proper functionality. There is a variety of experimental evidence which describes this mechanism of functional loss and related diseases (118,119).

Binding sites: The abundance of binding sites can be seen from both numbers of types of functional sites and numbers of sites (Supplementary Table S1b and Table 1). A total of 113 758 sites belonging to 1105 subtypes of binding sites were collected. There are several studies on how binding sites are affected by nsSNVs and how they are linked to diseases (120,121). This study provides a comprehensive view of how germline and somatic mutations affect such sites. Out of 113 758 experimentally verified binding sites reported on the human proteome, 18 681 are affected by nsSNVs. Note that 12 286 of those impacted binding sites are replaced by nsSNVs not in dbSNP, the remaining 6395 are mutated by nsSNVs found in dbSNP. Similar impact patterns can be seen (Tables 2a and 2b and Figure 2) between binding sites and active sites, however, binding sites are overrepresented with an abundance of residue-changing variants (Table 2a; *P*-value 4.81E-32). Further analysis between somatic and germline nsSNVs provides a similar profile as observed for active site variations: somatic variation is overrepresented (*P*-value of 2.26E-110) while germline variations are underrepresented (*P*-value of 7.86E-20). The top six binding sites that are affected are DNA binding site, ATP binding site, Ca2+ binding site, dimer interface, substrate binding site and Cytokine receptor motif.

#### Pathway and GO analysis summary

There are recent studies that report the association and coordination between different types of PTMs from both experimental and computational aspects (29,31,122–125). GO overrepresentation analysis is often used in gene expression analysis and a variety of other large-scale studies (31,126–128). However, there is no effort yet dedicated to the comprehensive study of the effects of germline and somatic variations on all major PTM sites. The GO term enrichment test and the follow-up clustering analysis among 10 main types of functional sites can be seen in Figure 3a. A total of 332 distinct GO terms of biological process, molecular function and cellular components showed over-/underrepresentation to at least one type of nsSNV affected functional site. It is interesting to see ubiquitylation and acetylation are grouped together by similarity of pathway hits as similar observations about functional associations are found in literature (31). We also see nsSNV affected phosphorylation, N-glycosylation, enzyme active sites and protein binding sites are enriched/depleted significantly among a wide range of GO terms with distinct patterns. Phosphorylation, especially, behaves similar to what is described in previous studies (31,129) and is more likely to be involved in protein interaction networks and pathways. Besides GO, PANTHER pathway analysis provided similar clustering results. Note that 249 distinct pathways were found to be influenced by proteins containing nsSNV-affected functional sites. Similar patterns and relationship between acetylation and ubiquitylation can be observed in Figure 3b. Details of the significantly influenced pathways and GO terms are described in the above section. It is important to note that GO and pathway annotations are different ways of classifying genes, and they have different coverage and focus and therefore can only reflect the current knowledge available to the scientific community. Maturation of methods in metabolic modeling is expected to provide additional insights into how these variations effects the biological system (130).

### Proteome-wide pan-cancer analysis

**Figure 4.**
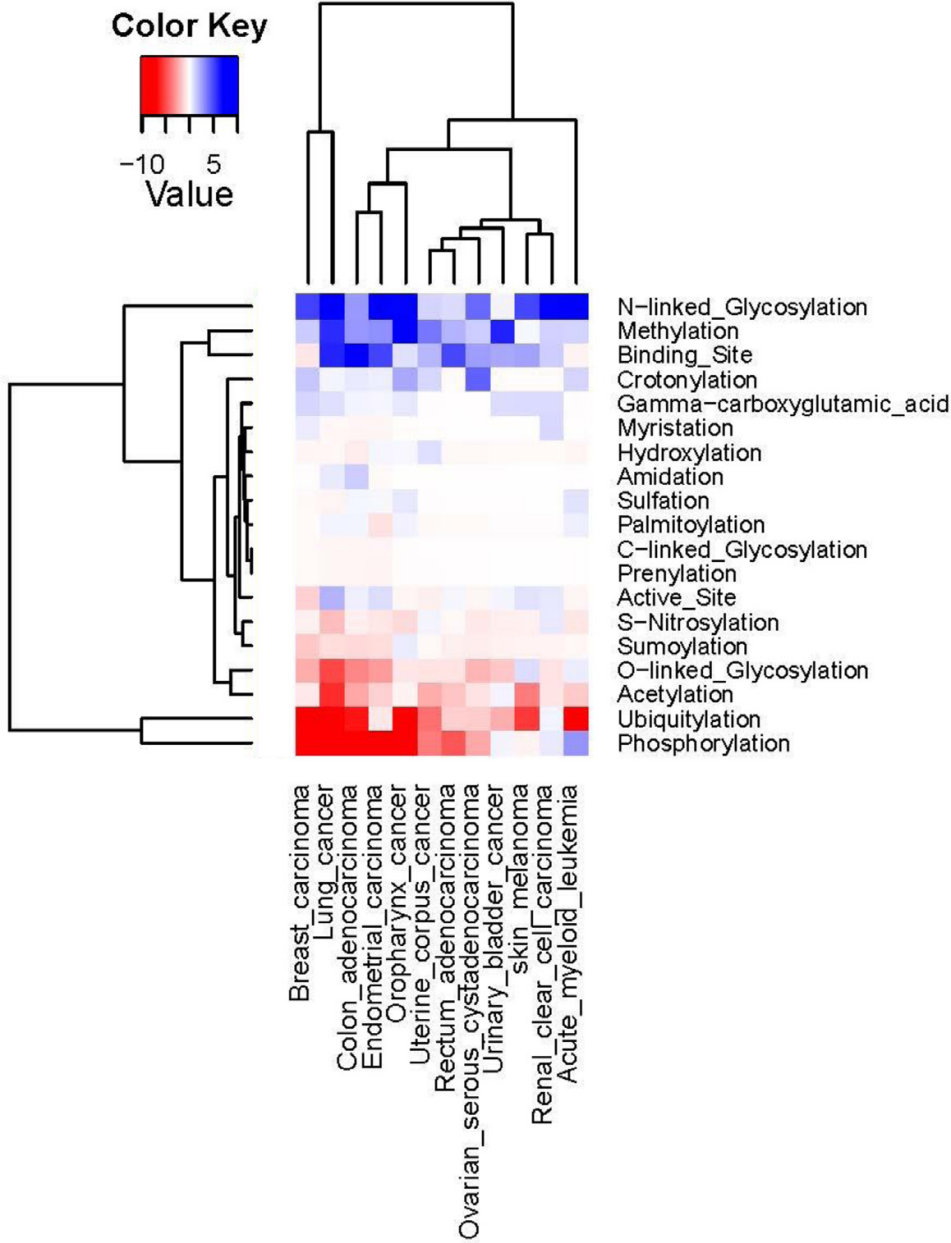
Pan-cancer analysis of cancer associated nsSNVs. Underrepresentation is colored in red and overrepresentation is in blue. The darkness of the color in each box reflects the absolute value of –log(*P*-value). Based on the patterns of –log(*P*-value), functional sites and cancer types are clustered into different groups. In the heatmap, functional sites are listed, with the most underrepresented type of nsSNV at the bottom and most overrepresented nsSNV at the top.

Comparative analysis of the functional effects of variations on different cancer types provides a comprehensive overview of the similarities and differences between different cancer types (Figure 4, Supplementary Table S4b). In order to better understand the functional impact of mutations in different cancer types we undertook a proteome-wide pan-cancer analysis. The systematic pan-cancer scale investigation of somatic mutations has been impossible to achieve until recent advances of sequencing technologies (131,132). Recently, the TCGA pan-cancer analysis project across 12 tumor types has been launched (133) and several others are ongoing. Recent studies by Alexandrov *et al.* (131) reported genome-wide mutational signatures and regions. A follow-up study by Jia *et al.* (41) have shown that for nine cancers, 3–5 independent mutational signatures in each cancer underlie each cancer genome where both mutagen exposure and changes in DNA repair systems were identified as key mutagenesis forces. Other previous works have shown that there exists specific mutational patterns in different cancers (134,135) and recent studies have started identifying genes to be ‘truly associated’ with cancer (23,43,135–136). Our pan-cancer study goes a step forward by identifying not just the genes but key mutations that potentially affect known protein function.

Our data set of cancer-associated nsSNVs was mapped to 73 DO terms (24). The DO terms were further collapsed into 12 DO terms to facilitate pan-cancer analysis (Figure 4, functional site affecting mutation counts can be found in Supplementary Table S4a and *P*-value is available in Supplementary Table S4b). The mapping result from cancer types to our total nsSNV data set (1 705 285) resulted in the assignment of 573 789 nsSNVs associated with at least one cancer type. Note that 38 549 of these nsSNVs affected a protein functional site and 13 159 nsSNVs have cancer associations. We found that across the different cancer types, N-linked glycosylation, methylation and binding sites are all preferentially affected by somatic mutation. Conversely, phosphorylation and ubiquitylation are significantly less impacted by variation compared to what is expected based on the distribution of all variations. O-linked glycosylation and acetylation show a similar trend albeit less significant. It is interesting to note that unlike most of the cancer types having statistically less nsSNVs interrupting phosphorylation sites, acute myeloid leukemia (DOID:9119) shows overrepresentation of cancer nsSNVs on phosphorylation sites (*P*-value 1.24E-03). It has been shown that phosphorylation status regulates the function of CCAAT/enhancer-binding protein alpha, a crucial factor associated with the development of various subtypes of acute myeloid leukemia (AML) (137). Furthermore, a recent NGS study on AML clinical samples revealed that 59% of case samples harbor at least one nsSNV in signaling genes which are pathogenesis related (138). However, further research is required to elucidate the role of phosphorylation on the development of AML.

Comparison across cancer types show that lung cancer (DOID:1324) and breast carcinoma (DOID:3459) are clustered together (Figure 4) as nsSNVs from both cancer types showing underrepresentation of phosphorylation, ubiquitylation and O-linked glycosylation and overrepresentation in N-linked glycosylation and methylation. Interestingly, for active sites, the two cancer types behave differently. Other cancer types such as urinary bladder cancer (DOID:11054), ovarian serous cystadenocarcinoma (DOID:5746), rectum adenocarcinoma (DOID:1996) and uterine corpus cancer (DOID:9460) are grouped together since they share similar mild or lack of significance for both groups of functional sites that commonly show over- (like N-linked glycosylation) and underrepresentation (such as phosphorylation). Skin melanoma (DOID:8923) and renal clear cell carcinoma (DOID: 4467) are grouped together because they share the overrepresentation of nsSNVs abolishing N-linked glycosylation site and normal occurrence of nsSNVs on phosphorylation site, which is usually underrepresented. The nsSNVs from endometrial carcinoma (DOID: 2871) and colon adenocarcinoma (DOID: 234) share the pattern of overrepresentation on binding sites and underrepresentation for O-linked glycosylation sites compared to oropharynx cancer (DOID: 8857) which is clustered alone beside the clade of the former two. In terms of overrepresentation on phosphorylation sites, nsSNV from acute myeloid leukemia (DOID: 9119) is separated on one branch. It is interesting to note that the ontologically closely related cancer types from DO hierarchy are not clustered into the same branch but into adjacent branches as they show different functional impact patterns. For instance, colon adenocarcinoma is not clustered together with rectum adenocarcinoma, but they are all under the term colorectal cancer (DOID:9256) in DO. Similar patterns can be seen in uterine corpus cancer and endometrial carcinoma.

It has been suggested that the number of driver mutations required during oncogenesis is relatively small, so it is possible to identify such driver mutations by looking to see which mutations are shared across cancer types (43). Potential driver mutations were identified based on mutations that are present across multiple cancer types. We successfully assigned 5496 distinct genes with 13 132 functional site affecting nsSNVs to cancer types. Out of these 5496 genes, 514 genes with 755 nsSNV affected functional sites are associated with two or more cancers (full list in Supplementary Table S4c). And 51 of these genes harboring 106 nsSNV affected functional sites were found in three or more cancer types.

**Figure 5.**
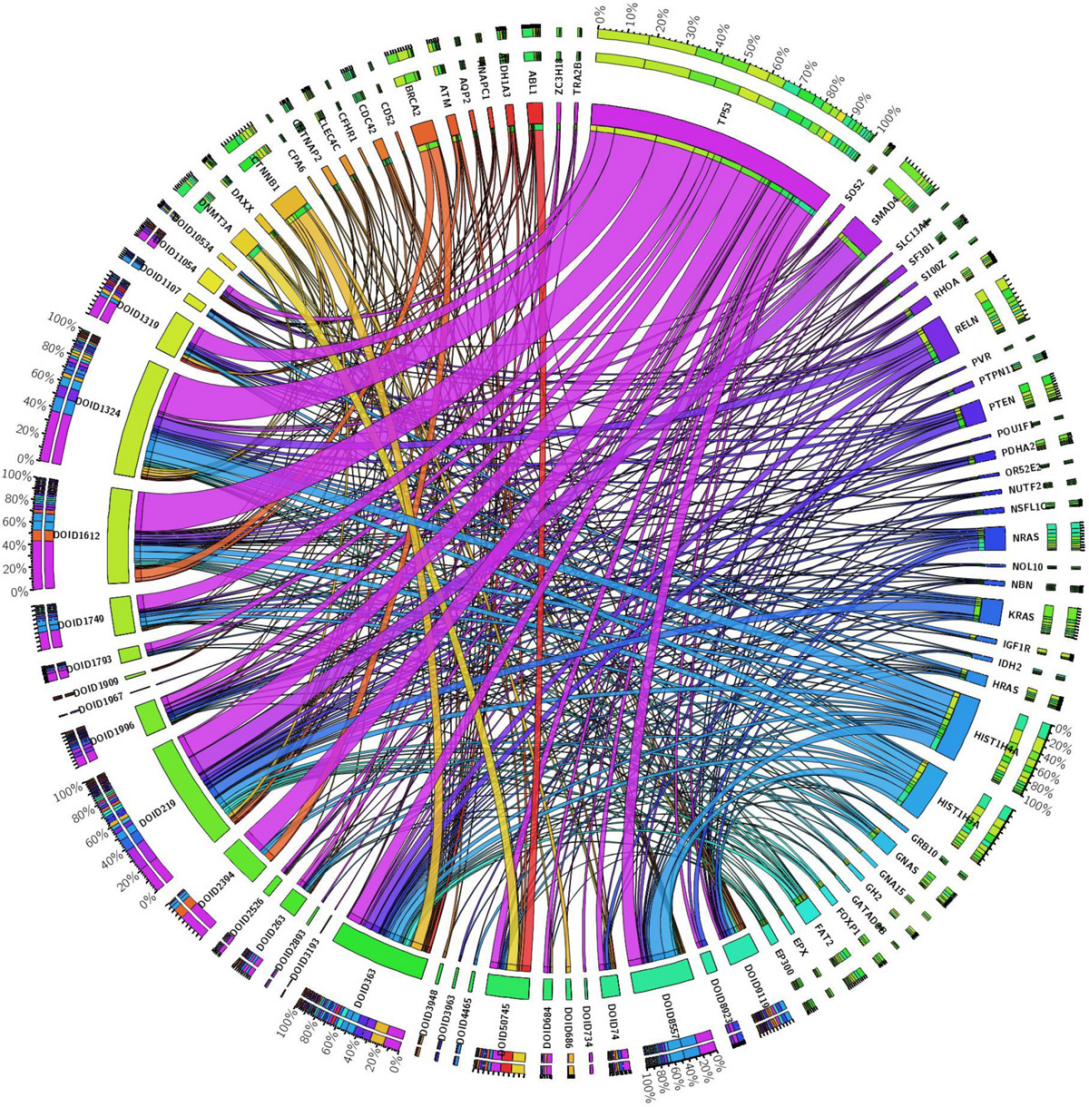
Circos plot representing 990 cancer-associated mutations from 51 genes that contain at least one somatic mutation from at least two or more cancer types. Cancer types are listed using DO identifiers. Each gene in the plot is assigned a unique color and the ribbon width indicates the corresponding counts of total cancer-associated mutations for that gene. DO identifier terms refer to following cancer types: DOID:8923 = skin melanoma, DOID:1319 = brain cancer, DOID:363 = uterine cancer, DOID:684 = hepatocellular carcinoma, DOID:1749 = squamous cell carcinoma, DOID:263 = kidney cancer, DOID:50745 = diffuse large B-cell lymphoma, DOID:2893 = cervix carcinoma, DOID:8557 = oropharynx cancer, DOID:1996 = rectum adenocarcinoma, DOID:3948 = adrenocortical carcinoma, DOID:2526 = prostate adenocarcinoma, DOID:3193 = peripheral nerve sheath neoplasm, DOID:1107 = esophageal carcinoma, DOID:686 = liver carcinoma, DOID:1793 = pancreatic cancer, DOID:3963 = thyroid carcinoma, DOID:1324 = lung cancer, DOID:219 = colon cancer, DOID:1612 = breast cancer, DOID:2394 = ovarian cancer, DOID:1909 = melanoma, DOID:1967 = leiomyosarcoma, DOID:11054 = urinary bladder cancer, DOID:734 = urethra cancer, DOID:10534 = stomach cancer, DOID:9119 = acute myeloid leukemia, DOID:74 = hematopoietic system disease, DOID:4465 = papillary renal cell carcinoma.

In order to better understand what we found through this pan-cancer analysis, a comparison was performed by using the gene list of 127 significantly mutated genes (SMG) identified by a recent pan-cancer study that used TCGA data (43). By mapping the published SMG data it was found that 88 out of 127 SMGs have a functional site affecting mutation based on our analysis. Out of these 88 genes, 29 were found in our list of 514 genes affected in two or more cancers described above. From our study we wish to emphasize 13 genes which are (i) present in the list of 127 SMG set, (ii) present in our list of 51 genes which are present in three or more cancers and (iii) have key functional site-affecting mutations. Table 3 provides a list of these genes and mutations. While the majority of these 13 genes are well known to be involved in cancer, this work highlights for the first time, key point mutations through a comprehensive pan-cancer analysis. To better visualize the data we plotted all functional site-affecting cancer-associated mutations from the above 51 key genes using Circos plot (Figure 5, the matrix seen Supplementary Table S4d). The plot includes a total of 990 mutations related to one or more cancers (106 of them are associated with three or more cancer types). Functional site-affecting mutations from genes like TP53, HIST1H4A, HIST1H3A, RELN, SMAD4, CTNN81, DICER1, KRAS, NRAS, BRCA2 and PTEN account for majority of the nsSNVs associated with cancers. Figure 5 provides an overview of distinct patterns on how different functional site-disrupting nsSNVs are contributed by each gene. The 127 SMGs consists of one miRNA (MIR142) and 126 protein coding genes. Note that 21 of them either are found to have no functional site annotations (such as EGR3) or have functional site(s) which do not overlap with with nsSNVs (e.g. PCBP1). Note that 17 of them contain at least one nsSNV affected functional site, which links to disease annotation(s) (e.g. MECOM) but not a cancer type; and the rest 88 of them harbor one or more mutations associated with at least one cancer type. For those proteins which have functional sites and nsSNVs in our data set but show no overlap between the two can point to passenger mutations or might indicate paucity of known/annotated functional sites. For example, PCBP1, which has around 40 functional sites and 38 nsSNVs on a 356 amino acids long sequence, shows surprisingly no overlap of nsSNVs and functional sites. It is possible that for such proteins either are protected from mutation of functional sites or the knowledge of all of the functional sites in the protein is incomplete.

**Table 3.** Key mutations identified through pan-cancer analysis

Gene Name	UniProtAC	Variation	Functional site	Cancer type	Conserv.	PDB ID
TP53	P04637	K164E	Acetylation	ovarian cancer; brain cancer; colon cancer; lung cancer	Yes	3D06
ATM	Q13315	N2875S	Binding Site(ATP binding site)	breast cancer; rectum adenocarcinoma; colon cancer	Yes	N/A
TP53	P04637	R248Q	Binding Site(DNA binding site)	brain cancer; lung cancer; uterine cancer; colon cancer; rectum adenocarcinoma; breast cancer; ovarian cancer; kidney cancer	Yes	3D06
TP53	P04637	R248W	Binding Site(DNA binding site)	brain cancer; lung cancer; uterine cancer; colon cancer; rectum adenocarcinoma; breast cancer; ovarian cancer	Yes	3D06
TP53	P04637	S241F	Binding Site(DNA binding site)	breast cancer; ovarian cancer; uterine cancer	Yes	3D06
TP53	P04637	S241C	Binding Site(DNA binding site)	breast cancer; uterine cancer; kidney cancer	Yes	3D06
TP53	P04637	A276P	Binding Site(DNA binding site)	breast cancer; ovarian cancer; hepatocellular carcinoma	Yes	3D06
TP53	P04637	C277F	Binding Site(DNA binding site)	urinary bladder cancer; ovarian cancer; breast cancer; lung cancer	Yes	3D06
TP53	P04637	R273H	Binding Site(DNA binding site)	brain cancer; lung cancer; uterine cancer; colon cancer; rectum adenocarcinoma; breast cancer; ovarian cancer	Yes	3D06
TP53	P04637	R273L	Binding Site(DNA binding site)	breast cancer; ovarian cancer; lung cancer	Yes	3D06
TP53	P04637	R273P	Binding Site(DNA binding site)	breast cancer; ovarian cancer; lung cancer	Yes	3D06
TP53	P04637	R273C	Binding Site(DNA binding site)	brain cancer; lung cancer; uterine cancer; colon cancer; rectum adenocarcinoma; breast cancer; ovarian cancer; hematopoietic system disease; acute myeloid leukemia	Yes	3D06
TP53	P04637	R273S	Binding Site(DNA binding site)	uterine cancer; diffuse large B-cell lymphoma; lung cancer (found in ‘-’ strand)	Yes	3D06
DNMT3A	Q9Y6K1	R792H	Binding Site (substrate interaction site)	acute myeloid leukemia; hematopoietic system disease; diffuse large B-cell lymphoma; lung cancer	Yes	2QRV
TP53	P04637	C275Y	Binding Site(DNA binding site)	brain cancer; lung cancer; rectum adenocarcinoma; colon cancer; breast cancer; ovarian cancer; pancreatic cancer	Yes	3D06
TP53	P04637	N239S	Binding Site(DNA binding site)	diffuse large B-cell lymphoma; lung cancer; uterine cancer; colon cancer; breast cancer; ovarian cancer; kidney cancer	Yes	3D06
TP53	P04637	R280T	Binding Site(DNA binding site)	breast cancer; oropharynx cancer; lung cancer; urinary bladder cancer	Yes	3D06
TP53	P04637	R280I	Binding Site(DNA binding site)	ovarian cancer; colon cancer; lung cancer	Yes	3D06
TP53	P04637	R280G	Binding Site(DNA binding site)	hematopoietic system disease; oropharynx cancer; lung cancer	Yes	3D06
KRAS	P01116	Q61H	Binding Site(GEF interaction site)	rectum adenocarcinoma; pancreatic cancer; colon cancer; uterine cancer	Yes	4LUC
KRAS	P01116	Q61L	Binding Site(GEF interaction site)	uterine cancer; colon cancer; lung cancer	Yes	4LUC
NRAS	P01111	Q61L	Binding Site(GEF interaction site)	brain cancer; colon cancer; lung cancer	Yes	3CON
NRAS	P01111	Q61R	Binding Site(GEF interaction site)	rectum adenocarcinoma; uterine cancer; colon cancer; breast cancer; ovarian cancer; hematopoietic system disease; acute myeloid leukemia	Yes	3CON
NRAS	P01111	Q61K	Binding Site(GEF interaction site)	lung cancer; rectum adenocarcinoma; colon cancer; uterine cancer; hematopoietic system disease; acute myeloid leukemia	Yes	3CON
NRAS	P01111	Q61H	Binding Site(GEF interaction site)	skin melanoma; hematopoietic system disease; acute myeloid leukemia	Yes	3CON
BRCA2	P51587	R3052W	Binding Site(OB2/OB3 interface)	breast cancer; ovarian cancer; colon cancer	Yes	N/A
BRCA2	P51587	R3052Q	Binding Site(OB2/OB3 interface)	breast cancer; ovarian cancer; uterine cancer	Yes	N/A
PTPN11	Q06124	Q510L	Binding Site (Substrate)	brain cancer; hematopoietic system disease; acute myeloid leukemia	Yes	3B7O
PTPN11	Q06124	Q510H	Binding Site (Substrate)	brain cancer; hematopoietic system disease; acute myeloid leukemia	Yes	3B7O
IDH2	P48735	R172S	Binding Site (Substrate)	brain cancer; acute myeloid leukemia; colon cancer	Yes	4JA8
DNMT3A	Q9Y6K1	C497Y	Binding Site(Zn binding site)	acute myeloid leukemia; hematopoietic system disease; diffuse large B-cell lymphoma	Yes	3A1B
SMAD4	Q13485	R361H	Binding Site(trimer interface)	oropharynx cancer; rectum adenocarcinoma; colon cancer; lung cancer	Yes	1YGS
TP53	P04637	C242Y	Binding Site(zinc binding site)	brain cancer; oropharynx cancer; lung cancer	Yes	3D06
TP53	P04637	H179R	Binding Site(zinc binding site)	brain cancer; lung cancer; uterine cancer; colon cancer; breast cancer; ovarian cancer; hematopoietic system disease; prostate adenocarcinoma; pancreatic cancer	Yes	3D06
TP53	P04637	H179Y	Binding Site(zinc binding site)	breast cancer; brain cancer; oropharynx cancer; lung cancer	Yes	3D06
TP53	P04637	C176Y	Binding Site(zinc binding site)	ovarian cancer; colon cancer; lung cancer	Yes	3D06
TP53	P04637	C176F	Binding Site(zinc binding site)	breast cancer; brain cancer; colon cancer; lung cancer	Yes	3D06
TP53	P04637	C238S	Binding Site(zinc binding site)	breast cancer; hematopoietic system disease; oropharynx cancer	Yes	3D06
TP53	P04637	C238F	Binding Site(zinc binding site)	brain cancer; urinary bladder cancer; lung cancer; uterine cancer; oropharynx cancer; breast cancer; ovarian cancer	Yes	3D06
TP53	P04637	C238Y	Binding Site(zinc binding site)	brain cancer; diffuse large B-cell lymphoma; lung cancer; uterine cancer; colon cancer; rectum adenocarcinoma; breast cancer; ovarian cancer; pancreatic cancer	Yes	3D06
TP53	P04637	R110L	Methylation	ovarian cancer; pancreatic cancer; lung cancer	No	3D06
TP53	P04637	R213L	Methylation	breast cancer; oropharynx cancer; colon cancer	Yes	3D06
TP53	P04637	R213Q	Methylation	kidney cancer; brain cancer; uterine cancer; urinary bladder cancer; rectum adenocarcinoma	Yes	3D06
EP300	Q09472	R580Q	Methylation	rectum adenocarcinoma; colon cancer; uterine cancer	Yes	N/A
TP53	P04637	R209I	Methylation	breast cancer; uterine cancer; squamous cell carcinoma	Yes	3D06
TP53	P04637	R337L	Methylation	colon cancer; oropharynx cancer; lung cancer	Yes	1A1E
TP53	P04637	R337C	Methylation	brain cancer; ovarian cancer; oropharynx cancer; breast cancer; colon cancer; hematopoietic system disease; acute myeloid leukemia	Yes	1A1E
TP53	P04637	S215R	Phosphorylation	breast cancer; ovarian cancer; hematopoietic system disease; squamous cell carcinoma	Yes	3D06
CTNNB1	P35222	S37F	Phosphorylation	stomach cancer; brain cancer; uterine cancer; lung cancer	Yes	3FQR
CTNNB1	P35222	S37C	Phosphorylation	urinary bladder cancer; uterine cancer; liver carcinoma; lung cancer	Yes	3FQR
CTNNB1	P35222	S33C	Phosphorylation	brain cancer; uterine cancer; liver carcinoma; colon cancer	Yes	3FQR
CTNNB1	P35222	S45F	Phosphorylation	liver carcinoma; uterine cancer; colon cancer; lung cancer	Yes	N/A
TP53	P04637	T155N	Phosphorylation	breast cancer; brain cancer; squamous cell carcinoma	No	3D06
TP53	P04637	T155P	Phosphorylation	breast cancer; pancreatic cancer; lung cancer	No	3D06
CTNNB1	P35222	T41A	Phosphorylation	uterine cancer; colon cancer; lung cancer	Yes	2G57
TP53	P04637	T211I	Phosphorylation	colon cancer; brain cancer; oropharynx cancer	Yes	3D06
PTEN	P60484	Y155C	Phosphorylation	breast cancer; uterine cancer; thyroid carcinoma	Yes	N/A
SF3B1	O75533	K700E	Ubiquitylation	breast cancer; diffuse large B-cell lymphoma; acute myeloid leukemia; pancreatic cancer; prostate adenocarcinoma	Yes	N/A
TP53	P04637	K164E	Ubiquitylation	ovarian cancer; brain cancer; colon cancer; lung cancer	Yes	3D06
TP53	P04637	K132N	Ubiquitylation	breast cancer; colon cancer; oropharynx cancer; urinary bladder cancer	Yes	3D06
TP53	P04637	K132E	Ubiquitylation	breast cancer; ovarian cancer; lung cancer	Yes	3D06
TP53	P04637	K132R	Ubiquitylation	ovarian cancer; colon cancer; lung cancer	Yes	3D06

In addition to the above analysis we wanted to interrogate the data to identify mutations that are reported in multiple studies in hopes of gaining a slightly different perspective of the genes and mutations associated in cancer and providing a measure of validation to our analysis of cancer-specific mutations. To achieve this we counted the number of publications associated with a specific variation (based on counting unique PubMed IDs). We found 3787 functional site-disrupting nsSNVs supported by two or more published studies (Supplementary Table S4e). Out of those 3787 mutations, 379 are described in three or more studies. Based on our review of the publications associated with the mutations we conclude that although there are several publications that associate cancer to specific mutations, very few of these mutations are reported in databases. Focused biocuration can connect cancer, mutation and PMIDs together which in turn can help identify mutations that have been identified by multiple studies.

#### Conservation analysis

**Figure 6.**
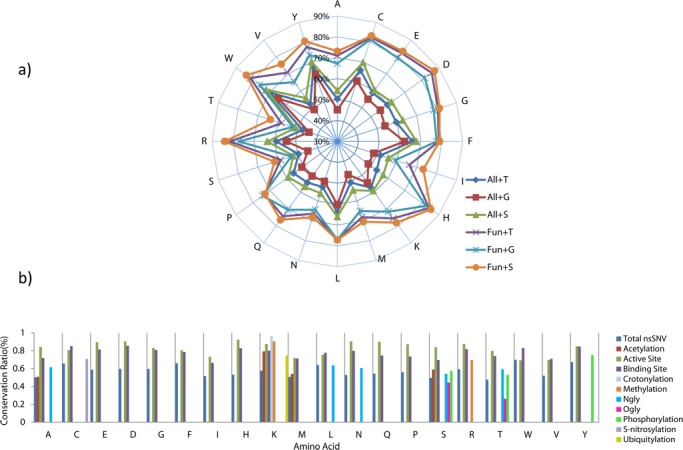
Conservation analsysis of nsSNVs. (a) Conservation ratio profile of germline/somatic variations and protein functional site affected by nsSNVs. The conservation ratio is calculated based on the percent of nsSNV impacted amino acids found to be conserved according to the BLAST results. In the radar chart, the radiating axis shows the conservation ratio values ranging from 0.3 to 0.9. ‘ALL’ stands for a specific type of amino acid that is affected by nsSNV regardless of functional site annotation. ‘Fun’ stands for an amino acid containing a functional site affected by nsSNV. ‘G’ stands for germline nsSNV site while ‘S’ stands for somatic nsSNV site. ‘T’ represents total conservation ratio for both germline and somatic nsSNVs. (b) Amino acid-based conservation ratio profile of various types of functional site affected by nsSNVs. The conservation ratio, which is calculated by the same method as in Figure 6a, is presented as the height of each bar. For each amino acid, depending on the type of functional site there can be one or multiple bar(s) that are colored differently. Each colored bar is represents the conservation ratio of a specific type of functional site affected by nsSNVs for that particular type of amino acid. For instance, the color ‘red’ is for acetylation, so there are red bars that occur among A(Ala), K(Lys), M(Met) and S(Ser).

Conservation analysis can be used as a complimentary method to measure the importance of a specific site from an evolutionary perspective. The conservation ratio ranges from 0 to 1 as described in Materials and Methods. We found that functional sites impacted by nsSNVs are significantly more conserved than nsSNV sites which are not associated with any known function (Supplementary Table S5a). Other than the significance analysis using *P*-value, the absolute ratio provides finer resolution (Figure 6a) and at the same time illustrates the distinct differences between germline and somatic SNVs. It is clear that somatic SNVs disrupts more conserved sites compared to germline mutations in both functional site affecting nsSNVs and total nsSNVs (All+S and Func+S, respectively, in Figure 6a). Generally, we found nearly all the sites affecting a functional site has a higher conservation ratio than the global nsSNV conservation ratio, especially binding site (on average 0.78) and active site (on average 0.83) as can be seen in Figure 6b. Common PTM types, like N-linked glycosylation and phosphorylation, have a mild conservation ratio which is higher than the global ratio for total nsSNVs. O-linked glycosylation is the only exception where the conservation ratio for nsSNV affecting the site has a value (Ser 0.44, Thr 0.26) even lower than the global nsSNV ratio (Ser 0.50, Thr 0.46). More experimentally verified O-glycosylation sites are needed to evaluate if this trend holds. Figure 6b only delineates an overall trend among different amino acids while Supplementary Table S5 provides the details of total conservation ratio, somatic/germline ratio on all the main amino acid grouped by different type of functional sites in our data set.

#### Example filtering and sorting of data to identify validation targets

There are several ways one can filter and sort the data to identify priority validation targets. In addition to the analysis mentioned above, we present below several examples of sorting the data to retrieve potential targets which can be further characterized and validated to connect genomic variation to functional impact and disease.

Through the comprehensive analysis of variations in all types of cancers that lead to loss of phosphorylation sites, we identified 143 mutations present in at least 2 types of cancers and 16 mutations found in 3 or more cancers (Supplementary Table S4c). From our analysis of TCGA breast tumor cancer samples (CSR porject (3)), we found a nsSNV (hg19 chr17:12915009) that changes reference nucleotide G to A. This mutation was found in 33% of the tumor samples and 12% of the control samples. The SNV maps to the protein zinc phosphodiesterase ELAC protein 2 (ELAC2; Q9BQ52) and changes 217 serine (S) to leucine (L). In UniProtKB, this mutation is recorded as a natural variant with the note ‘in HPC2; does not affect the enzymatic activity’. However, in our data set, we found this mutation is shared among CSR, NCI-60 (27 out of 60 cell lines (14)), dbSNP and annotated in UniProtKB to cause the loss of a phosphorylation receptor Ser (a phosphoserine site (35)). We also found that the site is conserved in 4 out of 5 orthologous proteins in mammals (for list of organisms see Materials and Methods). This mutation is linked to a SwissVar record for ‘prostate cancer, hereditary, 2’. This mapping and annotation bridges the gap between the phosphorylation site and disease-driven mutation and provides clues to test the possible mechanism of disease.

For loss of N-glycosylation sites we identified 63 mutations present in 2 or more types of cancers and 5 mutations found in 3 or more cancers (Supplementary Table S4c). Here we use the replacement of an asparagine residue in position 130 of SLCO1B1 protein (UniProtKB accession: Q9Y6L6) as an example. The genomic variation on hg19 chr12:21329738 from A to G is shared by CSR, ICGC, dbSNP and NCI-60 exome project. The variation can be mapped to UniProtKB entry Q9Y6L6 (protein name: Solute carrier organic anion transporter family member 1B1). We found that there is nearly a 20% frequency difference between CSR breast cancer samples and normal samples for this specific variation and the variation can be widely detected on 28 cell lines across the NCI-60 cell line panel. The corresponding protein site is under moderate conservation (conserved in 3 out of 5 orthologous proteins).

A comprehensive analysis of variations in all types of cancers that lead to loss of active sites identified 33 such mutations that are present in no less than 2 type of cancers and 1 mutation were found in 3 or more cancers (Supplementary Table S4c). The mutation of an enzyme's active site can destroy its activity. In the 2385 nsSNV modified active sites, 222 are annotated to be associated with diseases. Here we choose one variation not yet well-studied to identify disease associations for which we have a distinct sample frequency among the case and control samples of our CSR TCGA breast cancer research. The genomic variation on human genome hg19 chr14: 24707479 from G to A occur in 13% of case samples but only 4.8% of control samples of our CSR TCGA breast cancer data. The same variation has also been reported by the NCI-60 cell line panel exome study (5 cell lines out of total 60). The protein is GMP reductase 2 (GMPR2) and the variation is mapped to the protein's active site Gly on 242 which is changed to Asp by the nsSNV. This may cause loss of function resulting in a disease phenotype. It is interesting to note that it has been shown that lack of expression of the proteins GMPR2 and PPRA are associated with the basal phenotype and patient outcome in breast cancer (139) and loss of function may play a role in carcinogenesis. For loss of binding sites we identified 439 such mutations that are present in 2 or more cancer types and there are 19 mutations that can be seen across 4 and up to 9 distinct DO cancer terms (Supplementary Table S4c). As an example we highlight here a GTPase NRas (NRAS) protein and its variation in position 61 from Gln to Arg caused by genomic variation on hg19 chr1:115256529 from T to C. The SNV is present in TCGA, ICGC, IntOGen, NCI-60 cell line data set and dbSNP. The corresponding protein variation can also be found in the corresponding UniProtKB feature line. This binding site information is from the CDD database (26), which records the site as a part of GEF interacting site. General annotation in UniProtKB states that NRAS is activated by GEF. Thus, the mutated binding site of GEF interaction may lead to an impaired activation function of the protein, which can lead to the disease annotation for that site labeled ‘lung carcinoma cell and melanoma’ in SwissVar. By checking the reference (140) that identified the site as the disease-causing site, we found there is no explicit description how the disease is caused by the variation. The disrupted interaction with the activator GEF may serve as one possible explanation proposed by our study. Similar potential validation targets can be identified for all of the nsSNV-affected PTM and functional sites discussed in this paper.

### Phylogenetic classification of patients

**Figure 7.**
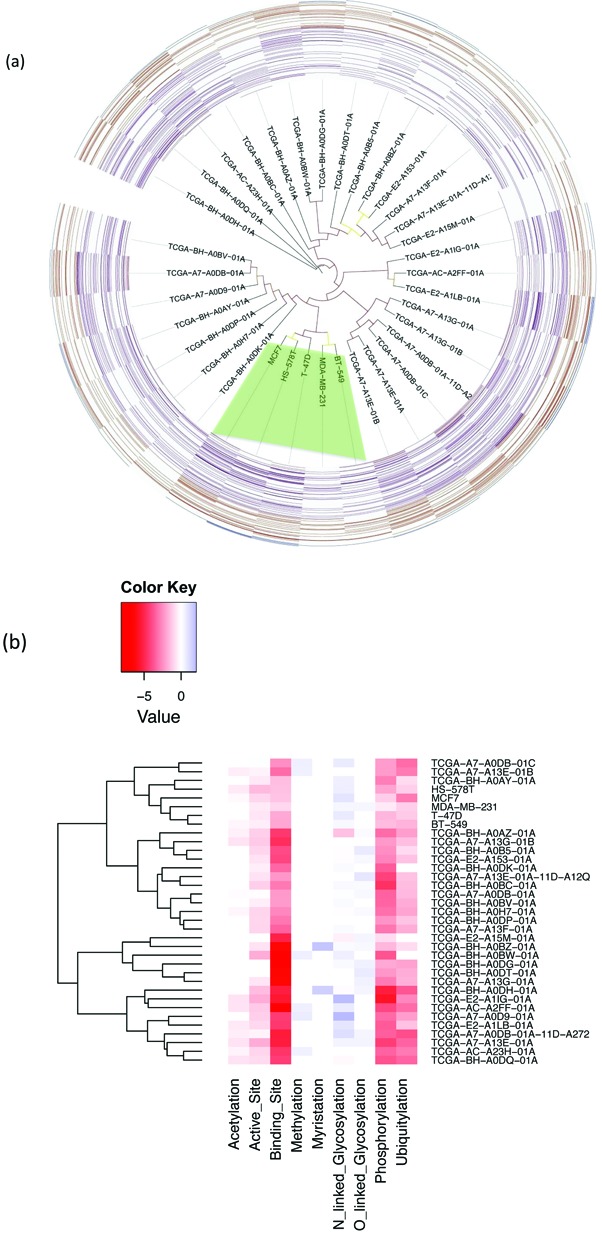
(a) The phylogenetic tree of the whole exome sequencing results of 30 TCGA breast cancer tumor samples (from 25 patients) and 5 NCI-60 breast cancer cell lines and the proteome-wide functional site analysis. The five breast cancer cell lines from the NCI-60 panel are shaded in green. Branches are colored to visualize the bootstrap value (color from red to yellow represents the corresponding bootstrap value from high to low). Heatmap demonstrates the status of loss of functional sites of each individual sample is overlaid at the tip of the phylogenetic tree branches. From inside to outside of the heatmap contains mutated functional sites from acetylation to ubiquitylation. (b) A hierarchal clustering is performed based on significance of mutational profile on functional site. Underrepresentation is colored in red and overrepresentation is in blue. The darkness of the color in each box reflects the absolute value of –log(*P*-value). Based on the patterns of –log(*P*-value), cancer samples/cell lines are clustered into different groups.

Cancers are primarily classified based on tissue of origin. Based on our pan-cancer analysis results described above we see that there is a need to develop additional methods of classification for biomarker discovery. Recently, we have developed a method that allows phylogenetic classification of tumor and normal samples based on mutation profile compared to the human reference genome (3). Such phylogenetic analysis promises a systematic view of multiple samples or a population which can help us to understand the background heterogeneity of each sample from patients with the same or similar disease phenotype. This analysis also facilitates personalized analysis which can lead to personalized diagnostics and therapeutics. In this study, we selected 30 breast cancer samples and compared them with 5 breast cancer derived NCI-60 cell lines. The cancer-centric phylogenetic tree in Figure 7a was generated based on SNVs extracted from the 35 samples (30 tumor samples and 5 cell lines). Phylogenetic analysis shows that the samples fall into few main groups and it is interesting to note that all NCI-60 cell lines, T-47D, HS-578T, MCF7, BT549 and MDA-MB-231 in the same clade with several patient samples associated with that specific branch. It has long been argued that breast cancer is a complex and heterogeneous disease. Till date several factors have been used to classify breast cancer types and cell lines that include expression analysis, histological type, tumour grade, lymph node status, presence of predictive markers such as oestrogen receptor and human epidermal growth factor receptor 2, luminal subtype, etc (141). The use of breast cancer cell lines to model breast cancer has benefits and we believe phylogenetic classification of the cell lines and tumor cells provides a higher level of classification that can complement existing methods and help investigate the functional impact of variation in different subgroups. Additionally, this type of analysis can also help provide some basic quality control step where multiple samples have been sequenced from the same patient from the same tissue. Figure 7a provides such an example where we can see that samples from the same tissue and same patient (share the same TCGA patient sample barcode) are paired in the same branch (TCGA-A7-A13E-01A and TCGA-A7-A13E-01B). It is important to remember that the phylogenetic tree is based on multiple sequence alignment of all exomes (patients and cell lines) and not just somatic mutations (3,19). If whole genome sequence data is present then based on the phyloSNP (82) algorithm one can classify the patients and the tumors with a higher resolution.

The merged matrix shows 527 nsSNVs that affect functional sites among 35 samples, which indicates overlapping of functional site impacting nsSNVs across samples. The heat map appended on the phylogenetic tree (Figure 7a) provides a visual representation of the distribution of those nsSNVs based on clustered samples. From inside to outside, the colored boxes represent acetylation, active site, binding site, methylation, N-linked glycosylation, O-linked glycosylation, other modified residue, phosphorylation and ubiquitylation.

These 527 nsSNVs impact nine different types of functional sites. The statistical significance of each type of nsSNV affected functional site from each sample was calculated and is presented as a heatmap (Figure 7b). Based on different patterns of significance, an unsupervised hierarchical clustering was performed to show the association between different samples. From the figure, acetylation, active site, binding site, phosphorylation site and ubiquitylation sites were found to have significant impact by nsSNVs. N-linked glycosylation and O-linked glycosylation present the overall trend of overrepresention of nsSNVs with few exceptions, which indicates the heterogeneity of functional profile from the genomic landscape of tumor samples. The sample level resolution also allows the observation of other outstanding data points. For example, there are few samples which show myristylation or methylation being affected by nsSNVs while the majority show no significance impact for these functional sites. When looking across the clustered samples, we can summarize that the grouped five cancer cell lines are relatively less impacted by nsSNVs in binding and phosphorylation sites than most of the TCGA patient samples, which suggests cell lines are under less selection or have higher tolerance to variations interrupting these types of functional sites. Comparing this clustering with SNV-based phylogenetic tree on the same group of samples (Figure 7a), additional insight can be gained. Shared result from the two figures includes the clustering of five cell lines. The fact that the cell lines are grouped together under the two distinct methods, suggests the differences of mutational profile and mutated functional profile between cell lines and patient samples are significant. Although, we agree to Meyerson view ‘millions and millions of people are getting cancer, and over time the statistical power of looking at primary tumors is going to be greater’(142); however, the boundary is sometimes bridged as can be seen in Figure 7b. There are three patient samples that cluster closely with the five cell lines as they share similar patterns of nsSNV affected functional sites. Rest of the samples are clustered in more or less different hierarchy than the SNV-based phylogenetic tree, indicating the heterogeneity of tumor samples. It is also interesting to see different mutated functional site profile among evolutionarily paired samples from the same tumor patient. Overall, we believe this type of analysis can provide an informative reference as a complement of traditional classification methodologies described above.

### Linking the nsSNV impacted functional sites to diseases

For this analysis disease associated SNVs from SwissVar, GWAS catalog project and dbSNP was collected. The collection resulted in 63 927 distinct entries though some cancer entries from different sources may overlap. Among 63 927, there are 2110 that are present in nsSNV affected functional sites (Supplementary Table S6, column Disvar). The top 10 genes which have the highest number of mutations (normalized based on their length) related to diseases are TP53, HBB, VHL, TTR, HMBS, HBA1, SOD1, GCH1, F8 and KRAS. Although, we attempted to have a comprehensive disease-related mutation list we realized that additional biocuration and literature mining-based methods can significantly increase this type of mapping. Increased mappings may lead to additional genes associated with nsSNVs and their impact to functional site.

## CONCLUSION

The significance of nsSNV impact calculated based on the source and type of functional site provides a comprehensive view of how germline and somatic variations are distributed on protein functional sites. Somatic nsSNVs collected from various cancer-centric databases, instead of showing random distribution, occurs on functional sites with significant over- or underrepresentation and therefore may be involved in the development of the tumor phenotype. Additionally, use of phylogenetic classification using SNVs and overlaying the tree with functional site variations provides a framework for novel translational and personalized medicine research and can lead to targeted diagnostics and therapeutics. It is important to note that many disease-related mutation sites are located in the non-coding regions of the genome (143). Analysis of those mutations using methods similar to the ones described in this paper may help to unravel some disease complexity. In this study even after collecting the most comprehensive information on protein functional sites we find only 2.29% affect the types of functional sites defined in our study. Therefore, the vast majority of cancer-associated mutations either have no effect on protein function or one can argue that our current knowledge of protein functional sites is highly limited. Additional protein functional site information in the coming years will help refine and improve this analysis. Our future plans include redoing the analysis described here every 2 years and compare and contrast the results over time.

It is also possible that neutral sequence variants may define individuals and their diseases (40). Additionally, certain disease phenotypes arise through combinations of many variants whose individual effects might not be damaging. With the development of sequencing technologies, high-quality genomics and proteomics data will help unravel the complexities of biological systems and help connect genomes to phenomes, especially for complex diseases like cancer and diabetes. In order to get a better understanding of the system, functional annotation that facilitates the exploration of the impact of a variant is necessary and hence active biocuration of genomic and proteomic data is of utmost important.

## SUPPLEMENTARY DATA

Supplementary Data are available at NAR Online.

SUPPLEMENTARY DATA

## References

[1] MacArthur D.G., Balasubramanian S., Frankish A., Huang N., Morris J., Walter K., Jostins L., Habegger L., Pickrell J.K., Montgomery S.B. (2012). A systematic survey of loss-of-function variants in human protein-coding genes. Science.

[2] Dingerdissen H., Motwani M., Karagiannis K., Simonyan V., Mazumder R. (2013). Proteome-wide analysis of nonsynonymous single-nucleotide variations in active sites of human proteins. FEBS J..

[3] Cole C., Krampis K., Karagiannis K., Almeida J.S., Faison W.J., Motwani M., Wan Q., Golikov A., Pan Y., Simonyan V. (2014). Non-synonymous variations in cancer and their effects on the human proteome: workflow for NGS data biocuration and proteome-wide analysis of TCGA data. BMC Bioinformat..

[4] Wu J., Li Y., Jiang R. (2014). Integrating multiple genomic data to predict disease-causing nonsynonymous single nucleotide variants in exome sequencing studies. PLoS Genet..

[5] Karagiannis K., Simonyan V., Mazumder R. (2013). SNVDis: a proteome-wide analysis service for evaluating nsSNVs in protein functional sites and pathways. Genom. Proteom. Bioinformat..

[6] Cooper D.N., Krawczak M., Polychronakos C., Tyler-Smith C., Kehrer-Sawatzki H. (2013). Where genotype is not predictive of phenotype: towards an understanding of the molecular basis of reduced penetrance in human inherited disease. Hum. Genet..

[7] Abunimer A., Smith K., Wu T.J., Lam P., Simonyan V., Mazumder R. (2014). Single-nucleotide variations in cardiac arrhythmias: prospects for genomics and proteomics based biomarker discovery and diagnostics. Genes (Basel).

[8] Hamosh A., Scott A.F., Amberger J.S., Bocchini C.A., McKusick V.A. (2005). Online Mendelian Inheritance in Man (OMIM), a knowledgebase of human genes and genetic disorders. Nucleic Acids Res..

[9] Landrum M.J., Lee J.M., Riley G.R., Jang W., Rubinstein W.S., Church D.M., Maglott D.R. (2013). ClinVar: public archive of relationships among sequence variation and human phenotype. Nucleic Acids Res..

[10] Mottaz A., David F.P., Veuthey A.L., Yip Y.L. (2010). Easy retrieval of single amino-acid polymorphisms and phenotype information using SwissVar. Bioinformatics.

[11] Lim D.H., Rehal P.K., Nahorski M.S., Macdonald F., Claessens T., Van Geel M., Gijezen L., Gille J.J., Giraud S., Richard S. (2010). A new locus-specific database (LSDB) for mutations in the folliculin (FLCN) gene. Hum. Mutat..

[12] Sherry S.T., Ward M.H., Kholodov M., Baker J., Phan L., Smigielski E.M., Sirotkin K. (2001). dbSNP: the NCBI database of genetic variation. Nucleic Acids Res..

[13] Abecasis G.R., Auton A., Brooks L.D., DePristo M.A., Durbin R.M., Handsaker R.E., Kang H.M., Marth G.T., McVean G.A., Genomes Project, C. (2012). An integrated map of genetic variation from 1,092 human genomes. Nature.

[14] Abaan O.D., Polley E.C., Davis S.R., Zhu Y.J., Bilke S., Walker R.L., Pineda M., Gindin Y., Jiang Y., Reinhold W.C. (2013). The exomes of the NCI-60 panel: a genomic resource for cancer biology and systems pharmacology. Cancer Res..

[15] Hudson T.J., Anderson W., Artez A., Barker A.D., Bell C., Bernabe R.R., Bhan M.K., Calvo F., Eerola I., International Cancer Genome, C. (2010). International network of cancer genome projects. Nature.

[16] Lawrence M.S., Stojanov P., Mermel C.H., Robinson J.T., Garraway L.A., Golub T.R., Meyerson M., Gabriel S.B., Lander E.S., Getz G. (2014). Discovery and saturation analysis of cancer genes across 21 tumour types. Nature.

[17] Nature Genetics Editorial. (2013). Taking pan-cancer analysis global. Nat. Genet..

[18] Bavarva J.H., McMahon W., Bavarva M.J., Karunasena E., Garner H.R. (2012). Standardizing next-generation sequencing experiments and analysis methods. Clin. Chem..

[19] Wu T.J., Shamsaddini A., Pan Y., Smith K., Crichton D.J., Simonyan V., Mazumder R. (2014). A framework for organizing cancer-related variations from existing databases, publications and NGS data using a High-performance Integrated Virtual Environment (HIVE). Database : J. Biol. Databases Curation.

[20] Lam P.V., Goldman R., Karagiannis K., Narsule T., Simonyan V., Soika V., Mazumder R. (2013). Structure-based comparative analysis and prediction of N-linked glycosylation sites in evolutionarily distant eukaryotes. Genom. Proteom. Bioinformat..

[21] Forbes S.A., Bindal N., Bamford S., Cole C., Kok C.Y., Beare D., Jia M., Shepherd R., Leung K., Menzies A. (2011). COSMIC: mining complete cancer genomes in the Catalogue of Somatic Mutations in Cancer. Nucleic Acids Res..

[22] UniProt Consortium. (2013). Update on activities at the Universal Protein Resource (UniProt) in 2013. Nucleic Acids Res..

[23] Gonzalez-Perez A., Perez-Llamas C., Deu-Pons J., Tamborero D., Schroeder M.P., Jene-Sanz A., Santos A., Lopez-Bigas N. (2013). IntOGen-mutations identifies cancer drivers across tumor types. Nat. Methods.

[24] Schriml L.M., Arze C., Nadendla S., Chang Y.W., Mazaitis M., Felix V., Feng G., Kibbe W.A. (2012). Disease Ontology: a backbone for disease semantic integration. Nucleic Acids Res..

[25] Li C.Y., Yu Q., Ye Z.Q., Sun Y., He Q., Li X.M., Zhang W., Luo J., Gu X., Zheng X. (2007). A nonsynonymous SNP in human cytosolic sialidase in a small Asian population results in reduced enzyme activity: potential link with severe adverse reactions to oseltamivir. Cell Res..

[26] Marchler-Bauer A., Lu S., Anderson J.B., Chitsaz F., Derbyshire M.K., DeWeese-Scott C., Fong J.H., Geer L.Y., Geer R.C., Gonzales N.R. (2011). CDD: a Conserved Domain Database for the functional annotation of proteins. Nucleic Acids Res..

[27] Hinz U. (2010). From protein sequences to 3D-structures and beyond: the example of the UniProt knowledgebase. Cell. Mol. Life Sci..

[28] Minguez P., Letunic I., Parca L., Bork P. (2013). PTMcode: a database of known and predicted functional associations between post-translational modifications in proteins. Nucleic Acids Res..

[29] Beltrao P., Bork P., Krogan N.J., van Noort V. (2013). Evolution and functional cross-talk of protein post-translational modifications. Mol. Syst. Biol..

[30] Cline M.S., Karchin R. (2011). Using bioinformatics to predict the functional impact of SNVs. Bioinformatics.

[31] Woodsmith J., Kamburov A., Stelzl U. (2013). Dual coordination of post translational modifications in human protein networks. PLoS Comput. Biol..

[32] Beltrao P., Albanese V., Kenner L.R., Swaney D.L., Burlingame A., Villen J., Lim W.A., Fraser J.S., Frydman J., Krogan N.J. (2012). Systematic functional prioritization of protein posttranslational modifications. Cell.

[33] Lu C.T., Huang K.Y., Su M.G., Lee T.Y., Bretana N.A., Chang W.C., Chen Y.J., Chen Y.J., Huang H.D. (2013). DbPTM 3.0: an informative resource for investigating substrate site specificity and functional association of protein post-translational modifications. Nucleic Acids Res..

[34] Dinkel H., Chica C., Via A., Gould C.M., Jensen L.J., Gibson T.J., Diella F. (2011). Phospho.ELM: a database of phosphorylation sites–update 2011. Nucleic Acids Res..

[35] Hornbeck P.V., Kornhauser J.M., Tkachev S., Zhang B., Skrzypek E., Murray B., Latham V., Sullivan M. (2012). PhosphoSitePlus: a comprehensive resource for investigating the structure and function of experimentally determined post-translational modifications in man and mouse. Nucleic Acids Res..

[36] Gupta R., Birch H., Rapacki K., Brunak S., Hansen J.E. (1999). O-GLYCBASE version 4.0: a revised database of O-glycosylated proteins. Nucleic Acids Res..

[37] Lee T.Y., Chen Y.J., Lu C.T., Ching W.C., Teng Y.C., Huang H.D., Chen Y.J. (2012). dbSNO: a database of cysteine S-nitrosylation. Bioinformatics.

[38] Li H., Xing X., Ding G., Li Q., Wang C., Xie L., Zeng R., Li Y. (2009). SysPTM: a systematic resource for proteomic research on post-translational modifications. Mol. Cell. Proteom..

[39] Peri S., Navarro J.D., Kristiansen T.Z., Amanchy R., Surendranath V., Muthusamy B., Gandhi T.K., Chandrika K.N., Deshpande N., Suresh S. (2004). Human protein reference database as a discovery resource for proteomics. Nucleic Acids Res..

[40] Bromberg Y., Kahn P.C., Rost B. (2013). Neutral and weakly nonneutral sequence variants may define individuality. Proc. Natl. Acad. Sci. U.S.A..

[41] Jia P., Pao W., Zhao Z. (2014). Patterns and processes of somatic mutations in nine major cancers. BMC Med. Genom..

[42] Editorial. (2013). Pan-cancer initiative finds patterns of drivers. Cancer Discovery.

[43] Kandoth C., McLellan M.D., Vandin F., Ye K., Niu B., Lu C., Xie M., Zhang Q., McMichael J.F., Wyczalkowski M.A. (2013). Mutational landscape and significance across 12 major cancer types. Nature.

[44] Reimand J., Wagih O., Bader G.D. (2013). The mutational landscape of phosphorylation signaling in cancer. Sci. Rep..

[45] Cline M.S., Craft B., Swatloski T., Goldman M., Ma S., Haussler D., Zhu J. (2013). Exploring TCGA Pan-Cancer data at the UCSC Cancer Genomics Browser. Sci. Rep..

[46] Weinstein J.N., Collisson E.A., Mills G.B., Shaw K.R., Ozenberger B.A., Ellrott K., Shmulevich I., Sander C., Stuart J.M., Cancer Genome Atlas ResearchNetwork (2013). The Cancer Genome Atlas Pan-Cancer analysis project. Nat. Genet..

[47] Reinhold W.C., Sunshine M., Liu H., Varma S., Kohn K.W., Morris J., Doroshow J., Pommier Y. (2012). CellMiner: a web-based suite of genomic and pharmacologic tools to explore transcript and drug patterns in the NCI-60 cell line set. Cancer Res..

[48] Gaudet P., Argoud-Puy G., Cusin I., Duek P., Evalet O., Gateau A., Gleizes A., Pereira M., Zahn-Zabal M., Zwahlen C. (2013). neXtProt: organizing protein knowledge in the context of human proteome projects. J. Proteome Res..

[49] Marchler-Bauer A., Bryant S.H. (2004). CD-Search: protein domain annotations on the fly. Nucleic Acids Res..

[50] Huang H., McGarvey P.B., Suzek B.E., Mazumder R., Zhang J., Chen Y., Wu C.H. (2011). A comprehensive protein-centric ID mapping service for molecular data integration. Bioinformatics.

[51] Altschul S.F., Gish W., Miller W., Myers E.W., Lipman D.J. (1990). Basic local alignment search tool. J. Mol. Biol..

[52] Chen C., Natale D.A., Finn R.D., Huang H., Zhang J., Wu C.H., Mazumder R. (2011). Representative proteomes: a stable, scalable and unbiased proteome set for sequence analysis and functional annotation. PloS ONE.

[53] Mi H., Thomas P. (2009). PANTHER pathway: an ontology-based pathway database coupled with data analysis tools. Methods Mol. Biol..

[54] Kiemer L., Bendtsen J.D., Blom N. (2005). NetAcet: prediction of N-terminal acetylation sites. Bioinformatics.

[55] Suo S.B., Qiu J.D., Shi S.P., Sun X.Y., Huang S.Y., Chen X., Liang R.P. (2012). Position-specific analysis and prediction for protein lysine acetylation based on multiple features. PloS ONE.

[56] Basu A., Rose K.L., Zhang J., Beavis R.C., Ueberheide B., Garcia B.A., Chait B., Zhao Y., Hunt D.F., Segal E. (2009). Proteome-wide prediction of acetylation substrates. Proc. Natl. Acad. Sci. U.S.A..

[57] Driscoll W.J., Mueller S.A., Eipper B.A., Mueller G.P. (1999). Differential regulation of peptide alpha-amidation by dexamethasone and disulfiram. Mol. Pharmacol..

[58] Chapman-Smith A., Cronan J.E. (1999). The enzymatic biotinylation of proteins: a post-translational modification of exceptional specificity. Trends Biochem. Sci..

[59] Tan M., Luo H., Lee S., Jin F., Yang J.S., Montellier E., Buchou T., Cheng Z., Rousseaux S., Rajagopal N. (2011). Identification of 67 histone marks and histone lysine crotonylation as a new type of histone modification. Cell.

[60] Stenflo J., Suttie J.W. (1977). Vitamin K-dependent formation of gamma-carboxyglutamic acid. Ann. Rev. Biochem..

[61] Price P.A., Fraser J.D., Metz-Virca G. (1987). Molecular cloning of matrix Gla protein: implications for substrate recognition by the vitamin K-dependent gamma-carboxylase. Proc. Natl. Acad. Sci. U.S.A..

[62] Hu L.L., Niu S., Huang T., Wang K., Shi X.H., Cai Y.D. (2010). Prediction and analysis of protein hydroxyproline and hydroxylysine. PloS ONE.

[63] Cheng X., Collins R.E., Zhang X. (2005). Structural and sequence motifs of protein (histone) methylation enzymes. Ann. Rev. Biophys. Biomol. Struct..

[64] Xu Y., Ding J., Huang Q., Deng N.Y. (2013). Prediction of protein methylation sites using conditional random field. Protein Peptide Lett..

[65] Bologna G., Yvon C., Duvaud S., Veuthey A.L. (2004). N-Terminal myristoylation predictions by ensembles of neural networks. Proteomics.

[66] Blom N., Sicheritz-Ponten T., Gupta R., Gammeltoft S., Brunak S. (2004). Prediction of post-translational glycosylation and phosphorylation of proteins from the amino acid sequence. Proteomics.

[67] Mitchell D.A., Vasudevan A., Linder M.E., Deschenes R.J. (2006). Protein palmitoylation by a family of DHHC protein S-acyltransferases. J. Lipid Res..

[68] Ren J., Wen L., Gao X., Jin C., Xue Y., Yao X. (2008). CSS-Palm 2.0: an updated software for palmitoylation sites prediction. Protein Eng. Design Selection.

[69] Wong Y.H., Lee T.Y., Liang H.K., Huang C.M., Wang T.Y., Yang Y.H., Chu C.H., Huang H.D., Ko M.T., Hwang J.K. (2007). KinasePhos 2.0: a web server for identifying protein kinase-specific phosphorylation sites based on sequences and coupling patterns. Nucleic Acids Res..

[70] Obenauer J.C., Cantley L.C., Yaffe M.B. (2003). Scansite 2.0: proteome-wide prediction of cell signaling interactions using short sequence motifs. Nucleic Acids Res..

[71] Blom N., Gammeltoft S., Brunak S. (1999). Sequence and structure-based prediction of eukaryotic protein phosphorylation sites. J. Mol. Biol..

[72] Maurer-Stroh S., Eisenhaber F. (2005). Refinement and prediction of protein prenylation motifs. Genome Biol..

[73] Doulias P.T., Greene J.L., Greco T.M., Tenopoulou M., Seeholzer S.H., Dunbrack R.L., Ischiropoulos H. (2010). Structural profiling of endogenous S-nitrosocysteine residues reveals unique features that accommodate diverse mechanisms for protein S-nitrosylation. Proc. Natl. Acad. Sci. U.S.A..

[74] Hess D.T., Matsumoto A., Kim S.O., Marshall H.E., Stamler J.S. (2005). Protein S-nitrosylation: purview and parameters. Nat. Rev. Mol. Cell Biol..

[75] Huang S.Y., Shi S.P., Qiu J.D., Sun X.Y., Suo S.B., Liang R.P. (2012). PredSulSite: prediction of protein tyrosine sulfation sites with multiple features and analysis. Anal. Biochem..

[76] Teng S., Luo H., Wang L. (2012). Predicting protein sumoylation sites from sequence features. Amino Acids.

[77] Radivojac P., Vacic V., Haynes C., Cocklin R.R., Mohan A., Heyen J.W., Goebl M.G., Iakoucheva L.M. (2010). Identification, analysis, and prediction of protein ubiquitination sites. Proteins.

[78] Thomas P.D., Campbell M.J., Kejariwal A., Mi H., Karlak B., Daverman R., Diemer K., Muruganujan A., Narechania A. (2003). PANTHER: a library of protein families and subfamilies indexed by function. Genome Res..

[79] Thomas P.D., Kejariwal A., Guo N., Mi H., Campbell M.J., Muruganujan A., Lazareva-Ulitsky B. (2006). Applications for protein sequence-function evolution data: mRNA/protein expression analysis and coding SNP scoring tools. Nucleic Acids Res..

[80] Mazumder R., Morampudi K.S., Motwani M., Vasudevan S., Goldman R. (2012). Proteome-wide analysis of single-nucleotide variations in the N-glycosylation sequon of human genes. PloS ONE.

[81] Krzywinski M., Schein J., Birol I., Connors J., Gascoyne R., Horsman D., Jones S.J., Marra M.A. (2009). Circos: an information aesthetic for comparative genomics. Genome Res..

[82] Faison W.J., Rostovtsev A., Castro-Nallar E., Crandall K.A., Chumakov K., Simonyan V., Mazumder R. (2014). Whole genome single-nucleotide variation profile-based phylogenetic tree building methods for analysis of viral, bacterial and human genomes. Genomics.

[83] Price M.N., Dehal P.S., Arkin A.P. (2010). FastTree 2–approximately maximum-likelihood trees for large alignments. PloS ONE.

[84] Letunic I., Bork P. (2011). Interactive Tree Of Life v2: online annotation and display of phylogenetic trees made easy. Nucleic Acids Res..

[85] Welter D., MacArthur J., Morales J., Burdett T., Hall P., Junkins H., Klemm A., Flicek P., Manolio T., Hindorff L. (2014). The NHGRI GWAS Catalog, a curated resource of SNP-trait associations. Nucleic Acids Res..

[86] Schafmeier T., Haase A., Kaldi K., Scholz J., Fuchs M., Brunner M. (2005). Transcriptional feedback of Neurospora circadian clock gene by phosphorylation-dependent inactivation of its transcription factor. Cell.

[87] Singh C.R., Curtis C., Yamamoto Y., Hall N.S., Kruse D.S., He H., Hannig E.M., Asano K. (2005). Eukaryotic translation initiation factor 5 is critical for integrity of the scanning preinitiation complex and accurate control of GCN4 translation. Mol. Cell. Biol..

[88] Lou Y., Yao J., Zereshki A., Dou Z., Ahmed K., Wang H., Hu J., Wang Y., Yao X. (2004). NEK2A interacts with MAD1 and possibly functions as a novel integrator of the spindle checkpoint signaling. J. Biol. Chem..

[89] Pawson T. (2004). Specificity in signal transduction: from phosphotyrosine-SH2 domain interactions to complex cellular systems. Cell.

[90] Radivojac P., Baenziger P.H., Kann M.G., Mort M.E., Hahn M.W., Mooney S.D. (2008). Gain and loss of phosphorylation sites in human cancer. Bioinformatics.

[91] Mort M., Evani U.S., Krishnan V.G., Kamati K.K., Baenziger P.H., Bagchi A., Peters B.J., Sathyesh R., Li B., Sun Y. (2010). In silico functional profiling of human disease-associated and polymorphic amino acid substitutions. Hum. Mutat..

[92] Park C., Zhang J. (2011). Genome-wide evolutionary conservation of N-glycosylation sites. Mol. Biol. Evol..

[93] Hicke L. (2001). Protein regulation by monoubiquitin. Nat. Rev. Mol. Cell Biol..

[94] Kim H., Chen J., Yu X. (2007). Ubiquitin-binding protein RAP80 mediates BRCA1-dependent DNA damage response. Science.

[95] Hammond-Martel I., Yu H., Affar el B. (2012). Roles of ubiquitin signaling in transcription regulation. Cell. Signal..

[96] Gill D.J., Clausen H., Bard F. (2011). Location, location, location: new insights into O-GalNAc protein glycosylation. Trends Cell Biol..

[97] Schjoldager K.T., Clausen H. (2012). Site-specific protein O-glycosylation modulates proprotein processing—deciphering specific functions of the large polypeptide GalNAc-transferase gene family. Biochimica et biophysica acta.

[98] Slawson C., Hart G.W. (2011). O-GlcNAc signalling: implications for cancer cell biology. Nat. Rev. Cancer.

[99] Hart G.W., Slawson C., Ramirez-Correa G., Lagerlof O. (2011). Cross talk between O-GlcNAcylation and phosphorylation: roles in signaling, transcription, and chronic disease. Ann. Rev. Biochem..

[100] Gill D.J., Tham K.M., Chia J., Wang S.C., Steentoft C., Clausen H., Bard-Chapeau E.A., Bard F.A. (2013). Initiation of GalNAc-type O-glycosylation in the endoplasmic reticulum promotes cancer cell invasiveness. Proc. Natl. Acad. Sci. U.S.A..

[101] Starheim K.K., Gevaert K., Arnesen T. (2012). Protein N-terminal acetyltransferases: when the start matters. Trends Biochem. Sci..

[102] Van Damme P., Hole K., Pimenta-Marques A., Helsens K., Vandekerckhove J., Martinho R.G., Gevaert K., Arnesen T. (2011). NatF contributes to an evolutionary shift in protein N-terminal acetylation and is important for normal chromosome segregation. PLoS Genet..

[103] Sadoul K., Boyault C., Pabion M., Khochbin S. (2008). Regulation of protein turnover by acetyltransferases and deacetylases. Biochimie.

[104] Kamita M., Kimura Y., Ino Y., Kamp R.M., Polevoda B., Sherman F., Hirano H. (2011). N(alpha)-Acetylation of yeast ribosomal proteins and its effect on protein synthesis. J. Proteom..

[105] Behnia R., Panic B., Whyte J.R., Munro S. (2004). Targeting of the Arf-like GTPase Arl3p to the Golgi requires N-terminal acetylation and the membrane protein Sys1p. Nat. Cell Biol..

[106] Hwang C.S., Shemorry A., Varshavsky A. (2010). N-terminal acetylation of cellular proteins creates specific degradation signals. Science.

[107] Greer E.L., Shi Y. (2012). Histone methylation: a dynamic mark in health, disease and inheritance. Nat. Rev. Genet..

[108] Liu H., Galka M., Mori E., Liu X., Lin Y.F., Wei R., Pittock P., Voss C., Dhami G., Li X. (2013). A method for systematic mapping of protein lysine methylation identifies functions for HP1beta in DNA damage response. Mol. Cell.

[109] Sayegh J., Webb K., Cheng D., Bedford M.T., Clarke S.G. (2007). Regulation of protein arginine methyltransferase 8 (PRMT8) activity by its N-terminal domain. J. Biol. Chem..

[110] Bedford M.T., Richard S. (2005). Arginine methylation an emerging regulator of protein function. Mol. Cell.

[111] Lewis P.W., Muller M.M., Koletsky M.S., Cordero F., Lin S., Banaszynski L.A., Garcia B.A., Muir T.W., Becher O.J., Allis C.D. (2013). Inhibition of PRC2 activity by a gain-of-function H3 mutation found in pediatric glioblastoma. Science.

[112] Hassler M.R., Egger G. (2012). Epigenomics of cancer - emerging new concepts. Biochimie.

[113] Foster M.W., Hess D.T., Stamler J.S. (2009). Protein S-nitrosylation in health and disease: a current perspective. Trends Mol. Med..

[114] Aranda E., Lopez-Pedrera C., De La Haba-Rodriguez J.R., Rodriguez-Ariza A. (2012). Nitric oxide and cancer: the emerging role of S-nitrosylation. Curr. Mol. Med..

[115] Martinez-Ruiz A., Lamas S. (2004). S-nitrosylation: a potential new paradigm in signal transduction. Cardiovas. Res..

[116] Abunimer A., Smith K., Wu T.-J., Lam P., Simonyan V., Mazumder R. (2014). Single-nucleotide variations in cardiac arrhythmias: prospects for genomics and proteomics based biomarker discovery and diagnostics. Genes.

[117] Cho D.H., Nakamura T., Fang J., Cieplak P., Godzik A., Gu Z., Lipton S.A. (2009). S-nitrosylation of Drp1 mediates beta-amyloid-related mitochondrial fission and neuronal injury. Science.

[118] Hartmann T., Terao M., Garattini E., Teutloff C., Alfaro J.F., Jones J.P., Leimkuhler S. (2012). The impact of single nucleotide polymorphisms on human aldehyde oxidase. Drug Metabol. Disposition Biol. Fate Chem..

[119] Riballo E., Doherty A.J., Dai Y., Stiff T., Oettinger M.A., Jeggo P.A., Kysela B. (2001). Cellular and biochemical impact of a mutation in DNA ligase IV conferring clinical radiosensitivity. J. Biol. Chem..

[120] Bond C., LaForge K.S., Tian M., Melia D., Zhang S., Borg L., Gong J., Schluger J., Strong J.A., Leal S.M. (1998). Single-nucleotide polymorphism in the human mu opioid receptor gene alters beta-endorphin binding and activity: possible implications for opiate addiction. Proc. Natl. Acad. Sci. U.S.A..

[121] Begovich A.B., Carlton V.E., Honigberg L.A., Schrodi S.J., Chokkalingam A.P., Alexander H.C., Ardlie K.G., Huang Q., Smith A.M., Spoerke J.M. (2004). A missense single-nucleotide polymorphism in a gene encoding a protein tyrosine phosphatase (PTPN22) is associated with rheumatoid arthritis. Am. J. Hum. Genet..

[122] Zhao S., Xu W., Jiang W., Yu W., Lin Y., Zhang T., Yao J., Zhou L., Zeng Y., Li H. (2010). Regulation of cellular metabolism by protein lysine acetylation. Science.

[123] Kim W., Bennett E.J., Huttlin E.L., Guo A., Li J., Possemato A., Sowa M.E., Rad R., Rush J., Comb M.J. (2011). Systematic and quantitative assessment of the ubiquitin-modified proteome. Mol. Cell.

[124] Danielsen J.M., Sylvestersen K.B., Bekker-Jensen S., Szklarczyk D., Poulsen J.W., Horn H., Jensen L.J., Mailand N., Nielsen M.L. (2011). Mass spectrometric analysis of lysine ubiquitylation reveals promiscuity at site level. Mol. Cell. Proteom..

[125] Choudhary C., Kumar C., Gnad F., Nielsen M.L., Rehman M., Walther T.C., Olsen J.V., Mann M. (2009). Lysine acetylation targets protein complexes and co-regulates major cellular functions. Science.

[126] Zhang S., Cao J., Kong Y.M., Scheuermann R.H. (2010). GO-Bayes: Gene Ontology-based overrepresentation analysis using a Bayesian approach. Bioinformatics.

[127] Zambon A.C., Gaj S., Ho I., Hanspers K., Vranizan K., Evelo C.T., Conklin B.R., Pico A.R., Salomonis N. (2012). GO-Elite: a flexible solution for pathway and ontology over-representation. Bioinformatics.

[128] Blake J.A., Dolan M., Drabkin H., Hill D.P., Li N., Sitnikov D., Bridges S., Burgess S., Buza T., McCarthy F. (2013). Gene Ontology annotations and resources. Nucleic Acids Res..

[129] Vinayagam A., Stelzl U., Foulle R., Plassmann S., Zenkner M., Timm J., Assmus H.E., Andrade-Navarro M.A., Wanker E.E. (2011). A directed protein interaction network for investigating intracellular signal transduction. Sci. Signal..

[130] Dingerdissen H., Weaver D.S., Karp P.D., Pan Y., Simonyan V., Mazumder R. (2014). A framework for application of metabolic modeling in yeast to predict the effects of nsSNV in human orthologs. Biol. Direct.

[131] Alexandrov L.B., Nik-Zainal S., Wedge D.C., Aparicio S.A., Behjati S., Biankin A.V., Bignell G.R., Bolli N., Borg A., Borresen-Dale A.L. (2013). Signatures of mutational processes in human cancer. Nature.

[132] Stratton M.R., Campbell P.J., Futreal P.A. (2009). The cancer genome. Nature.

[133] Weinstein J.N., Collisson E.A., Mills G.B., Shaw K.R., Ozenberger B.A., Ellrott K., Shmulevich I., Sander C., Stuart J.M. (2013). The Cancer Genome Atlas Pan-Cancer analysis project. Nat. Genet..

[134] Yeang C.H., McCormick F., Levine A. (2008). Combinatorial patterns of somatic gene mutations in cancer. FASEB J..

[135] Ciriello G., Miller M.L., Aksoy B.A., Senbabaoglu Y., Schultz N., Sander C. (2013). Emerging landscape of oncogenic signatures across human cancers. Nat. Genet..

[136] Tamborero D., Gonzalez-Perez A., Perez-Llamas C., Deu-Pons J., Kandoth C., Reimand J., Lawrence M.S., Getz G., Bader G.D., Ding L. (2013). Comprehensive identification of mutational cancer driver genes across 12 tumor types. Sci. Rep..

[137] Pabst T., Mueller B.U. (2007). Transcriptional dysregulation during myeloid transformation in AML. Oncogene.

[138] Cancer Genome Atlas Research, N. (2013). Genomic and epigenomic landscapes of adult de novo acute myeloid leukemia. N. Eng. J. Med..

[139] Baker B.G., Ball G.R., Rakha E.A., Nolan C.C., Caldas C., Ellis I.O., Green A.R. (2013). Lack of expression of the proteins GMPR2 and PPARalpha are associated with the basal phenotype and patient outcome in breast cancer. Breast Cancer Res. Treatment.

[140] Raybaud F., Noguchi T., Marics I., Adelaide J., Planche J., Batoz M., Aubert C., de Lapeyriere O., Birnbaum D. (1988). Detection of a low frequency of activated ras genes in human melanomas using a tumorigenicity assay. Cancer Res..

[141] Holliday D.L., Speirs V. (2011). Choosing the right cell line for breast cancer research. Breast Cancer Res..

[142] Beroukhim R., Mermel C.H., Porter D., Wei G., Raychaudhuri S., Donovan J., Barretina J., Boehm J.S., Dobson J., Urashima M. (2010). The landscape of somatic copy-number alteration across human cancers. Nature.

[143] Wang Z., Moult J. (2001). SNPs, protein structure, and disease. Hum. Mutat..

