# Supplementary material for: Human germline and pan-cancer variomes and their distinct functional profiles
Source: Nucleic Acids Res. 2014 Sep 17;42(18):11570–88. doi: 10.1093/nar/gku772 (PMC4191387; doi:10.1093/nar/gku772)
Supplement: SUPPLEMENTARY DATA [file supp_42_18_11570__index.html]

Human germline and pan-cancer variomes and their distinct functional profiles — Human germline and pan-cancer variomes and their distinct functional profiles — SUPPLEMENTARY DATA 

# Human germline and pan-cancer variomes and their distinct functional profiles

## SUPPLEMENTARY DATA

**Files in this Data Supplement:**

- SUPPLEMENTARY DATA
- SUPPLEMENTARY DATA
