# Supplementary material for: Human germline and pan-cancer variomes and their distinct functional profiles
Source: Nucleic Acids Res. 2014 Sep 17;42(18):11570–88. doi: 10.1093/nar/gku772 (PMC4191387; doi:10.1093/nar/gku772)
Supplement: SUPPLEMENTARY DATA [file supp_gku772_nar-01885-n-2014-File003.docx]

**Supplementary Table Legends**

**Supplementary Table 1.** Summary statistics of the datasets. A) Summary statistics of nsSNV data sources. B) Summary counts of functional site dataset.

**Supplementary Table 2.** Data source based analysis of statistical significance of nsSNV affected functional sites. A) p-value table of data source based statistical analysis. B) The –LOG(p-value) table of data source based statistical analysis. C-I) Individual calculation of p-value for different data sources.

**Supplementary Table 3.** Pathway and GO term enrichment analysis. A) Gene Ontology term enrichment analysis result matrix. Values in the table are –LOG(p-value). Positive value stands for over-representation and negative value means under-representation. B) Pathway term enrichment analysis result matrix. Values in the table are –LOG(p-value). Positive value stands for over-representation and negative –value means under-representation.

**Supplementary Table 4.** Pan-cancer analysis results. A) nsSNV counts in diverse cancer types. B) The –LOG(p-value) table of cancer type based analysis of statistical significance of nsSNV affected functional sites. C) Mutation list of functional sites affected by nsSNVs that are associated with 2 or more cancer types. D) Matrix of 51 key genes and the associated cancer types. Numbers represent counts of mutation from each gene. E) Mutation list of functional sites impacted by nsSNVs associated with 2 or more PMIDs.

**Supplementary Table 5.** Conservation analysis results. A) Statistical significance (p-value) of the conservation ratio between functional sites affected by nsSNVs compared to global nsSNVs. B–M). Individual tables of amino acid type-based conservation ratio amongst different types of nsSNV sites. Each table provides 3 sets of conservation ratio which are germline, somatic and the combination of the two. B) global nsSNV sites; C) global functional site affected by nsSNVs; D–M. specific functional sites affected by nsSNVs).

**Supplementary Table 6.** DisVar (disease variation) scanning results. A) Mutation list of functional sites affected by nsSNVs that are associated with at least one type of disease recorded in the DisVar database. For details on DisVar database see Materials and Methods. B) Disease mutation density in the genes.
